# Alcohol consumption in P301S mice accelerates gait impairments, modifies aggregation of pathological tau and alters microglia within the hippocampus

**DOI:** 10.1111/acer.70123

**Published:** 2025-08-04

**Authors:** Nicole M. Maphis, Dominic A. Furlano, Seth A. David, David N. Linsenbardt

**Affiliations:** Department of Neurosciences, School of Medicine, University of New Mexico Health Sciences Center, Albuquerque, New Mexico, USA

**Keywords:** alcohol, Alzheimer’s disease, gait, neuroinflammation, pathological tau

## Abstract

**Background:**

Excessive alcohol use has emerged as the strongest modifiable risk factor for the development of Alzheimer’s disease (AD), but the underlying neural mechanisms are only beginning to be understood. Recent preclinical work suggests that alcohol consumption may have an impact on many pathologies and phenomena crucial to the development and pathogenesis of AD. However, little attention has been focused on pure tauopathy models to closely examine tau pathogenesis and neuroinflammation within a voluntary alcohol exposure paradigm.

**Methods:**

We exposed a mouse model of pathological tau (pTau), P301S, to a voluntary alcohol paradigm known as drinking-in-the-dark (DID) for 21 days of voluntary daily alcohol consumption.

**Results:**

In P301S mice, moderate alcohol consumption contributed to gait disruptions, acceleration of pTau spread, and enhancement of damage-associated microglia.

**Conclusions:**

This work identifies key interactions between alcohol and AD-related phenotypes which set the stage for future investigation into the neurobiological mechanisms behind these interactions.

## INTRODUCTION

Alzheimer’s Disease (AD), one of the most devastating diseases of the twenty-first century, is currently the seventh leading cause of death in the United States, primarily impacting individuals aged 65 and older. Over 6.9 million people in this age group live with AD (Alzheimer’s Association Report, 2024). The 2020 Census revealed that 5.3% of adults 65–74, 13.8% of adults 75–84, and 34.6% of adults 85+ had an AD diagnosis, which also projects the number of people with clinical AD to increase from 7.0 million in 2025 to 13.85 million in 2060 (Alzheimer’s Association Report, 2024). Early symptoms of AD include memory impairment, namely episodic short-term memory, and other declines in cognitive abilities, such as problem-solving, judgment, and organizational skills (Alzheimer’s Association Report, 2024). Two key pathologies implicated in memory and cognitive impairments in AD are extracellular amyloid beta (Aβ) plaques and intraneuronal pathological tau (pTau), as neurofibrillary tangles (NFTs) (Knopman et al., 2021). Clinicopathological studies suggest that AD pathology could develop at least 10–15 years before clinical symptoms appear (Tarawneh & Holtzman, 2012), highlighting a critical period during which environmental or behavioral factors may influence disease progression.

Sporadic AD, which represents a majority (95%) of cases, is a progressive neurological disorder that can be impacted by several factors. Nonmodifiable risk factors like age (Guerreiro & Bras, 2015), sex (Beam et al., 2018), and genetics (Andrews et al., 2023) are all thought to contribute to the lifelong risk of AD. However, recent meta-analyses highlight excessive alcohol use as a modifiable risk factor increasing AD risk (Livingston et al., 2020). In a report evaluating millions of electronic health records, alcohol use disorder (AUD) was identified as *the* strongest predictor of dementia, conferring a fourfold elevated risk compared with other modifiable risk factors (Schwarzinger et al., 2018). Furthermore, heavy drinking (8+ drinks/week) was found to predict more rapid cognitive decline in subjects already diagnosed with AD over a six-year follow-up (Heymann et al., 2016). Heavy drinking has also been indicated in shortening telomers, an indicator of advanced aging (Topiwala et al., 2022), suggesting that alcohol could accelerate brain aging. Additionally, as little as one drink a day could contribute to a reduction in gray and white matter in the brain (Daviet et al., 2022). Alcohol use among those 65+, particularly among women, has been on the rise over the last 20 years (Breslow et al., 2017). Thus, while an important link between alcohol and AD has emerged, the neural mechanisms mediating this link remain to be fully elucidated and are the focus of current ongoing work (Anton et al., 2025).

Microtubule-associated protein tau (MAPT) is essential for stabilizing microtubules within neurons (Mazanetz & Fischer, 2007). Under physiological conditions, its function is regulated by the phosphorylation at up to 80 sites (Noble et al., 2013). In AD, however, pTau accumulates to levels approximately three times higher than physiological tau (Wang et al., 2013). Some studies report that pTau is more predictive of AD onset (Binette et al., 2022) and correlates better with the progression of AD (Teunissen et al., 2022) compared with amyloid pathology. Evidence suggests that pTau originates from the entorhinal cortex (EC), prior to spreading to functionally connected brain regions, like the dentate gyrus (DG) (Mufson et al., 2016). Tauopathies—neurodegenerative diseases characterized by pathological tau aggregates, like NFTs and pTau—include AD as the most prevalent type. Thus, understanding how alcohol use and misuse could contribute to AD through tau-mediated pathogenesis remains an area of critical importance.

Rodent models of alcohol consumption are frequently used to investigate the neural consequences of alcohol exposure. One such model, drinking-in-the-dark (DID), is particularly useful in studying voluntary alcohol consumption because it reliably results in blood ethanol concentrations (BECs) of 80 mg/dL or higher. This model has also been useful for characterizing frontloading behavior, which we (Maphis et al., 2022) and others (Ardinger et al., 2022) have previously reported as a proxy for an animal’s willingness to experience alcohol’s post-absorptive effects.

Rodent models are also central to investigating and understanding the molecular mechanisms of AD. Popular models like P301S (aka PS19) and 3xTg have all been instrumental in decoding how Aβ plaques and pTau pathology both develop and interact with/induce neuroinflammation as well as contribute to other cognitive and behavioral disturbances. Recent evidence using a 3xTg-AD mouse model, which has mutations in both APP (amyloid precursor protein) and MAPT (Oddo et al., 2003), has provided some evidence that excessive alcohol may increase intraneuronal Aβ (Anton et al., 2024; Barnett et al., 2022; Day et al., 2023) and pTau (Tucker et al., 2022) either through neuroinflammation (Barnett et al., 2022; Qin et al., 2021) and/or autophagy dysregulation (Tucker et al., 2022). However, a clear need still exists to determine the neurobiological mechanisms by which alcohol alters the development and progression of AD. The goal of this work was to test the hypothesis that alcohol consumption during early adulthood would lead to alterations in daily home cage locomotor activity, circadian rhythms, and gait, due to underlying changes in pTau accumulation and neuroinflammation.

## METHODS

### Animals

Twenty-four (12 Male [M]/12 Female [F]) B6;C3-Tg (Prnp-MAPT*P301S)PS19Vle/J, Tau P301S (Yoshiyama et al., 2007), JAX #008169 and 24 (12 M/12 F) Noncarrier (nontransgenic, nTg, B6C3F1/J, JAX#100010) littermates were purchased from The Jackson Laboratory at postnatal (PND) Day 42. Upon arrival, animals were weighed and single-housed in standard Allentown shoebox mouse cages with amber walls, and a polyacrylic lid with predrilled air holes for constant video home cage monitoring. All 48 animals were placed in their location in pods of 4. Each shelf in our animal room was counter-balanced to house eight mice and include each sex (M/F), genotype (nTg/P301S), and fluid [Ethanol (EtOH)/Water (WAT)] for all groups. Coat color was carefully considered and segregated as equally as possible. Animals were acclimated for 21 days prior to any experimental manipulation.

**FIGURE 1:**
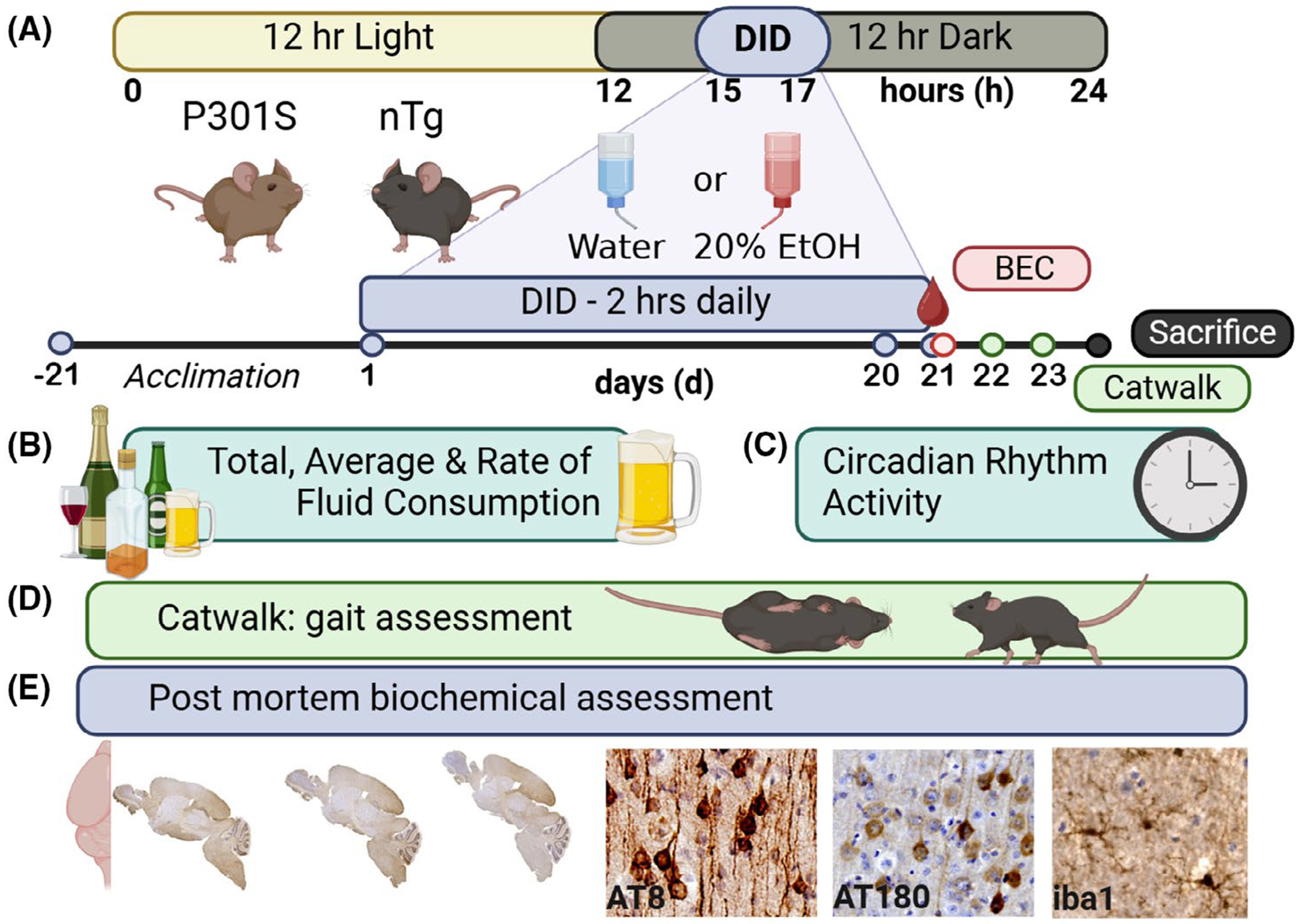
DID alcohol exposure model and timeline of experimental methods. (A) Animals maintained on a 12 h light/dark cycle were given limited access (2 hour, h) to either water (WAT) or a 20% ethanol solution (in water, EtOH) 3 h into the dark cycle. P301S (a tauopathy line) and their isogenic nontransgenic (nTg) littermates were acclimated to single housing, diet, and 12 h light/dark cycle for a period of 21 days prior to DID at postnatal day 60. DID continued for 21 days (D). On the last day of drinking, periorbital blood was taken for blood ethanol concentration (BEC). (B) Two-hour total fluid consumption, including average amount of fluid consumed and rate of consumption, was monitored for 21 days. (C) Roughly, 23.4 h of home cage activity was monitored to assess circadian rhythm for Days 2–20. (D) Two days after the final DID session, gait was assessed using Catwalk, and then, brain tissue was harvested. The right hemisphere was fixed/sectioned/processed for (E) pTau (AT8, AT180) and microglia (iba1). The other hemisphere was flash frozen for future genomic analysis. *Created in BioRender. Maphis, N. (2024)* https://BioRender.com/z73c527.

Mice were maintained on a reverse light/dark cycle, with lights off at 10:30 a.m. Experimenters were only in the room prior to and at the conclusion of every DID session to place and remove sipper tubes, respectively. Since we have previously reported on the influence of diet on binge-like behavior, animals were also switched over to the LD01 chow (LabDiet 5001, Purina) (Maphis et al., 2022) upon arrival to the facility. Cages were changed 1 day prior to the beginning of the experiment to eliminate diet as a factor in alcohol consumption. Mice were maintained, ad libitum, on LabDiet 5001 (LD01), which was placed on the cage floor to minimally obstruct video monitoring. Mice had access to animal resource facility (ARF) water, except during the 2-h DID session (Figure 1A,B).

### Home cage monitoring

Twelve Logitech, Carl Zeiss Tessar HD 1080 P cameras were used to record 23.4-h video sessions of home cage locomotion and behavior; one camera recorded four cages simultaneously. The center point of each animal was used to plot Cartesian XY location data through ANY-maze (Stoelting Co., Wood Dale, IL, USA), and data were exported as the distance traveled (centimeters, cm) per 10-s time bin. Three 23.4-h baseline video recordings were captured prior to the beginning of the experiment and then daily throughout all 21 DID sessions to plot home cage locomotor activity (LMA).

### Circadian rhythm activity

Exported files from ANY-maze were converted to .awd files and then analyzed through ClockLab (ActiMetrics, Lafayette, IN, USA) to assess circadian rhythm (Figure 1C). Home cage LMA, that is, distance traveled, was used as a proxy for activity. Notably, Days 1 and 21 were excluded from assessment since 23.4 h were not fully recorded on those dates. For Day 1, video tracking did not start until the beginning of the DID period, which means that the beginning of the dark cycle was not captured. For Day 21 (the last experimental day) experimenters were in the room for an exceptional amount of time to change cages, collect blood, and weigh animals, all of which disrupt normal behavior. Thus, circadian data were assessed for 19 days. Periods of time when animals were motionless were used to infer sleep and were used to calculate “sleep bouts.” Two additional nonparametric tools that were used to assess circadian rhythmicity—interdaily stability (IS) and intradaily variability (IV) were calculated. IS, a measure of how consistent the daily activity patterns are from 1 day to the next, is a 24-h value derived from the chi-square periodogram, which is normalized by the number of data points. The data can range from 0 (Gaussian noise) to 1 (highly stable). The final metric assessed for rhythmicity of the circadian cycle is IV, which is a measure of rhythm fragmentation, that is, the number of transitions between rest and activity on each day, where a value of 0 represents a perfect cycle (i.e., a sinusoidal wave), and a value of 2.0 represents random Gaussian noise or completely disjointed rest/active transitions.

### Drinking-in-the-dark (DID)

Mice were acclimated for 21 days to the DID room, single-housed conditions, light cycle, food, and volumetric drinking system noise; then, DID was conducted. DID, as performed here, has been previously described elsewhere in detail (Maphis et al., 2022); briefly, 3 h into their dark cycle, 50-mL bottles containing water from the Animal Research Facility (ARF) were replaced with either water (WAT) or 20% ethanol in ARF water (Alcohol, EtOH) with specialized sippers connected to the volumetric drinking monitor (VDM) fluid dispensing system, capable of monitoring fluid consumption in real time for a period of 2 h (Figure 1A). VDM sippers and VDM software (Columbus instruments) were used to record the number of sips, amount of fluid consumed in microliters (μL) per sip, and pattern of consumption within 10-s bins across a 2-h DID session (i.e., 720 bins) (Figure 1B). This was repeated daily for 21 days. nTg (*n* = 24) and P301S (*n* = 24) aged PND 62 were exposed to DID (WAT or EtOH) for 2 h daily for a period of 21 days, such that the final group size was *n* = 6 per sex, genotype, fluid to factor in *sex as a biological variable*. The last day of DID was omitted in the analyses due to the nature of the last day of the experiment, that is, experimenters were in the room for an extended period of time and EtOH access was protracted due to the collection of blood samples from the periorbital sinus for BEC analysis.

### Blood collection for blood ethanol concentration (BEC)

For the quantification of BEC, periorbital sinus blood was drawn immediately following the last day of DID, on Day 21 (Figure 1A). Blood samples were allowed to clot at room temperature for a period of 2 h, before being centrifuged at 15,000 RPM for 15 min to isolate plasma. Plasma was stored at −20°C until enzymatic assessment of BECs could be conducted using an Analox Ethanol Analyzer (Analox Instruments, Lunenburg, MA). One animal died prior to blood collection, and so, BEC for this animal was estimated from the interpolated data of the linear regression between BECs and EtOH consumed on that day (please see Figure S1 for additional details).

### Gait assessment

Gait assessment was performed, as described previously (Jacquez et al., 2020), using the Catwalk XT (Noldus, Leesburg, VA, USA, Version 10.5/10.6) (Figure 1D). The ceiling of the CatwalkXT^®^ runway is illuminated by red light, which allows the silhouette of the mouse to be captured. Green light enters the glass runway and is internally reflected; therefore, light escapes each time a paw makes contact with the surface. Paw prints and silhouettes are recorded with a digital high-speed video camera under the catwalk. Animals walk freely across the illuminated runway in both directions for a minimum of 3 compliant runs per day for 2 days; noncompliant trials were excluded automatically by Noldus CatwalkXT^®^ software. Additional trials that were compliant but in which the animal turned around mid-run, paused for longer than 1 s, or reared more than once were excluded during the classification step by a trained/blinded technician. All remaining compliant trials were evaluated for each subject by averaging at least three runs per animal. Animals were tested twice on the catwalk, once 24 h after DID, and a second time 48 h following DID. Only the second day’s gait values were analyzed due to acclimation effects observed in previous studies. Catwalk XT 10.5 and 10.6 software was used to analyze the data. Significant differences were defined as those that survived FDR-correction with *q* = 0.10.

### Euthanasia

After 2 days of catwalk assessment, mice were sacrificed to collect brain tissue. Briefly, animals were administered a sublethal dose of ketamine prior to being transcardially perfused with ice-cold phosphate-buffered saline (PBS). Following perfusion, the brain was carefully extracted, hemi-sected; one half of the brain (right) was drop fixed into a solution of 4% Paraformaldehyde (PFA)/PBS for post fixation (Immunohistochemistry [IHC]) while the other half (left) was snap frozen in liquid nitrogen, stored at −80°C for future RNAseq analysis.

### Immunohistochemistry (IHC)

Right hemi-brains were postfixed in 4%PFA for 24 h, then cryopreserved in 30% Sucrose/PBS for at least 24 h. Following cryoprotection, brains were embedded in optimal cutting temperature (OCT; TissueTek, Sakura, #4583) with liquid nitrogen. Embedded hemi-brains were stored in −20°C for at least 24 h before sectioning at 30 μm using a cryostat (Cryostar NX50; Thermo Fisher). Free-floating sections were stored in 24-well plates containing PBS and stored at 4°C until processed.

Brain tissue was processed to assess the protein expression of pTau and to characterize microglial phenotype across eight regions of interest using standard IHC procedures (Figure 1E). Briefly, free-floating brain sections were washed with PBS and then PBST (0.01% Triton/PBS), and then incubated in a blocking solution containing 5% normal goat sera (Abcam) in 0.4% PBS-Triton X-100. Primary antibodies were diluted at 1:500 in blocking solution (overnight at 4°C). After primary antibody incubation, sections were washed and then incubated for 1 h with avidin–biotin complex (ABC, Vectastain Elite^®^ABC Kit, Vector Laboratories, PK-6100) at a dilution of 1:100 in 0.1% PBST for 1 h. Following additional washes, signal was developed using 3,3′-Diaminobenzidine (DAB, SIGMAFAST^™^, Sigma, D4293-50SET), prepared in distilled water. DAB development was stopped with a 5-min wash in distilled water. Tissue sections were carefully affixed to Tissue Path Superfrost Plus Gold Microscope Slides (Thermo Fisher, 15-188-48) using paint brushes and allowed to dry completely. Once dry, slides were counterstained with Harris' Modified Hematoxylin (Thermo Fisher, SH30) for 1 min, rinsed under tap water for 5 min, and then dehydrated through a series of escalating ethanol concentrations. Finally, slides were cleared with two incubations in xylene and glass coverslips (#1, CORNING 22 × 50 mm, 2975) were adhered with Permount mounting medium (Thermo Fisher, SP-15). Detailed information on antibodies, dilution, species, manufacturer, and catalog number is in Table S1.

### HALO^®^ analysis of IHC for pTau accumulation

Whole-mount sagittal sections were scanned in at 20× using the Axioscan digital slide scanner (Zeiss, Germany) and analyzed using the HALO software (Indica Labs^®^, Corrales, New Mexico, USA). In order to assess pathological tau (pTau) accumulation, AT8 and AT180-stained slides were quantified using the HALO^®^ Area Quantification (v.2.4.3) settings (Irwin et al., 2015). Briefly, the default algorithm was applied to a region of interest while analysis fine-tuning was conducted under 1× magnification for optimal settings of weak, moderate, and strongly stained signals. Once trained on a few slides, the HALO classifier algorithm was run by a masked to treatment investigator who manually confirmed the output for each section and ROI across 24 P301S samples (CA1, CA2, CA3, dentate gyrus [DG], Cortex [CX], Retrosplenial Cortex [RSC], Subiculum [SUB], Hindbrain). Using HALO digital quantification settings for AT8 and for AT180 (detailed in Table S2) data captured were as follows: Percent (%) Positive (+) tissue, % Weakly+ tissue, % Moderately + tissue, % Strongly + tissue, and Average Positive Optical Density (OD) for both AT8 and AT180. OD is proportional to the amount of stain present, so a higher OD indicates a greater amount and intensity of stain, while a lower OD represents a lower amount of stain and/or an increased diffusivity of stain.

### HALO^®^ analysis of IHC for microglia morphology

Whole-mount sagittal sections were scanned in at 20× using the Axioscan digital slide scanner (Zeiss, Germany) and analyzed with the HALO image analysis platform (Indica Labs^®^, Corrales, New Mexico, USA). To assess microglia phenotype, DAB IHC slides probed with iba1 were first deconvolved using the HALO^®^ Deconvolution Algorithm (V. 1.1.8), which converted the iba1 signal into a pseudo-colored fluorescent green and the hemotoxylin counterstain into pseudo-colored fluorescent blue (DAPI). Microglia were then quantified using the HALO^®^ microglial activation FL module (v1.0.6) (Kloske et al., 2023). Analysis parameters were optimized under 1× magnification (Table S3). Eight regions of interest (ROI) were manually drawn across all 48 samples (CA1, CA2, CA3, dentate gyrus [DG], Cortex [CX], Retrosplenial Cortex [RSC], Subiculum [SUB], Hindbrain). To distinguish damage-associated microglia (DAMs) from homeostatic microglia (HoMs), an activation threshold of 1.5 was applied. So, fewer than 1.5 processes/cell body indicates a more ameboid/phagocytic phenotype (Kloske et al., 2023). Data for microglia analysis captured were as follows: Branchpoints/DAM, Endpoints/DAM, Branchpoints/HoM, and Endpoints/HoM. Branchpoints were automatically detected and marked with a pink pixel and were defined as any process that splits into at least two processes. The endpoints were marked with a cyan pixel and were defined as the termination of a process. Microglial analysis settings for the HALO^®^ Microglia Activation FL Module (v1.0.6) included Cell Body Diameter, Minimum (Min.) Cell Body Intensity, Min. Process Intensity, which were adjusted to accommodate background differences across sample batches (Table S3), whereas Max Process Radius, Max Fragmentation Length, and Activation Process Thickness were kept consistent across all 48 samples (Table S3). Settings within Nucleus Detection were kept consistent across all samples (Table S3).

### Statistical analyses

Drinking and home cage locomotor activity were evaluated using a combination of three-way repeated measures (RM) ANOVAs broken down on either sex or fluid, with follow-up analyses broken down on one or more factors. Circadian rhythm activity data were evaluated using three-way RM ANOVAs, with sex (M and F), genotype (nTg and P301S), and fluid type (EtOH or WAT) as between groups factors and day as within subjects factors. Follow-up analyses broken down on one or more factors were conducted based on the results of these ANOVAs. IHC protein quantification data for pTau was first evaluated using two-way ANOVAs based on the factors of sex and fluid since only P301S mice had pTau present, but IHC protein quantification data for iba1 was evaluated with three-way ANOVAs based on the factors of sex, genotype, and fluid type. Follow-up analyses broken down on one or more factors were conducted based on the results of these ANOVAs. Three-way ANOVAs were subjected to a Tukey’s post hoc testing, whereas two-way ANOVAs were subjected to uncorrected Fisher’s LSD post hoc testing. Catwalk/Gait data were evaluated using previously developed (Jacquez et al., 2020) methods implementing pairwise comparisons for groups of interest followed by false discovery rate (FDR) correction. Analyses were performed using Excel (Microsoft), Prism (GraphPad Prism, v 10.4.1 [627] for Windows, GraphPad Software, Boston, Massachusetts USA, www.graphpad.com), and MATLAB (MathWorks Inc. 2022). Optimization Toolbox version: 9.4 (R2022b), Natick, Massachusetts: The MathWorks Inc. https://www.mathworks.com.

## RESULTS

### Frontloading behavior only emerges within the P301S mice

**FIGURE 2:**
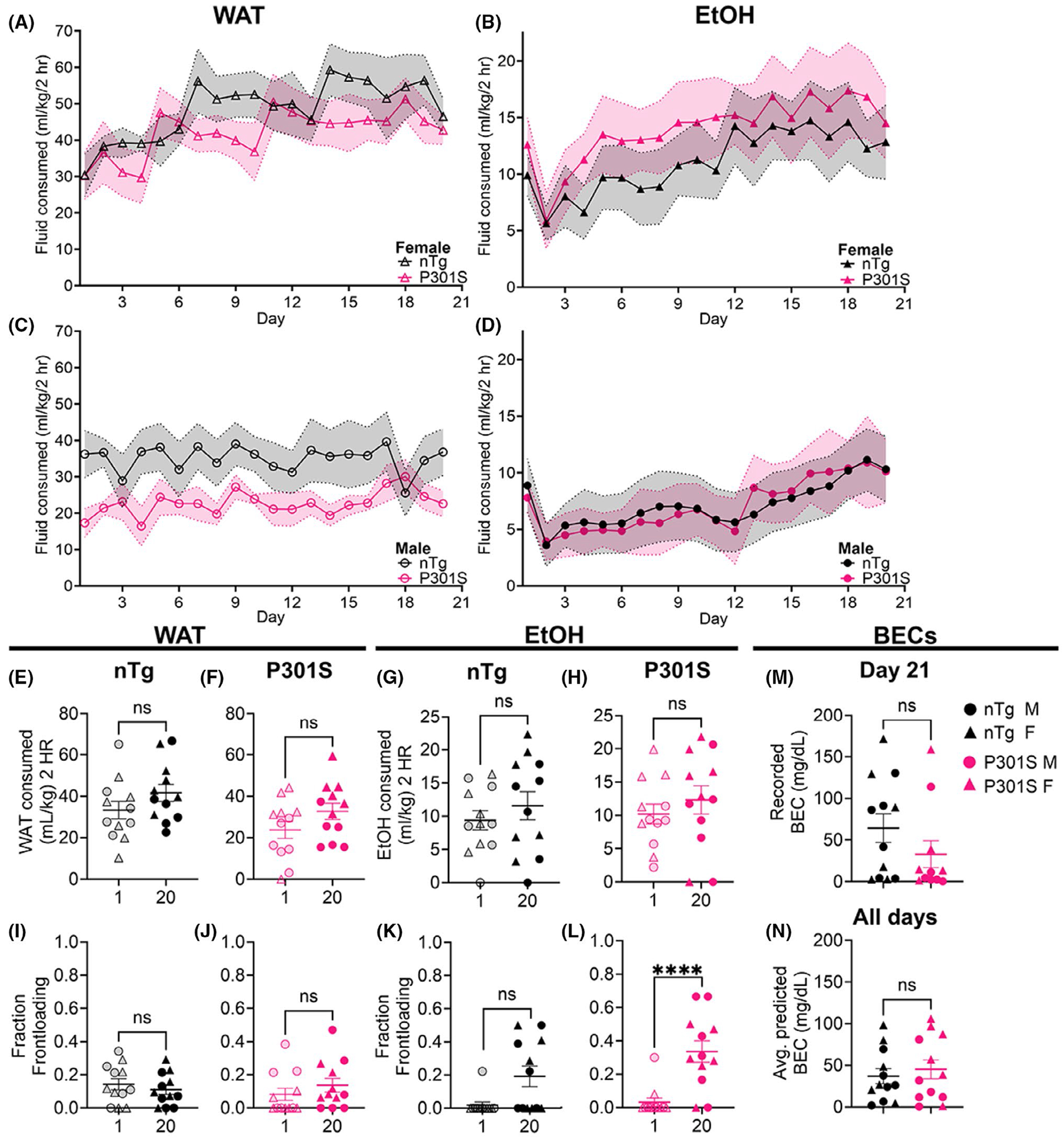
Water (WAT) and ethanol (EtOH) consumption patterns do not differ between nTg or P301S over a 21 days DID paradigm, but frontloading behavior develops only in EtOH-consuming P301S mice. (A) nTg (black) and P301S (pink) female mice consumed similar amounts of water (mL/kg) over each day of a 21 days DID experiment, but both strains increased consumption over 21 days DID. (B) nTg (black) and P301S (pink) female mice consumed similar amounts of EtOH over 21 days DID, escalating consumption at similar rates. (C) nTg (black) and P301S (pink) male mice consumed similar amounts of water (mL/kg) over each day of a 21 days DID experiment but did not escalate consumption over 21 days DID. (D) nTg (black) and P301S (pink) male mice consumed similar amounts of EtOH over 21 days DID, escalating consumption at similar rates. Amount of WAT consumed did not differ between D1 vs. D21 in (E) nTg or (F) P301S mice. Circle symbol = male, triangle symbol = female. Mean EtOH consumed did not differ on D1 vs. D20 between (G) nTg or (H) P301S. Circle symbol = male, triangle symbol = female. There is no frontloading behavior in (I) nTg or (J) P301S WAT-consuming mice, and it does not develop over the 21-day DID paradigm. Frontloading behavior does not significantly develop in EtOH-consuming nTg (K), but does in EtOH-consuming P301S (L) mice. (M)There was no significant difference in the blood ethanol concentration (BEC) measured in mg/dL between nTg and P301S mice. (N) There was no significant difference in the average predicted BEC over 20 days between nTg and P301S mice. For details on BEC calculation, please see Figure S1. *n* = 12 for each group: nTg WAT, nTg EtOH, P301S WAT, and P301S EtOH (6 M/6F per group). Significance stars featured are Tukey’s post hoc assessment of three-way ANOVAs. not significant; ns = > 30.05, **** = *p* < 0.0001.

The amount of fluid consumed in DID can be seen in Figure 2. Our initial interest was in assessing changes in drinking over all days, and as such evaluated the pattern of daily consumption in each sex separately. Separate analyses were conducted below that included sex as a factor. For females, the results of our three-way RM ANOVA with day as the within-subjects factor and fluid and genotype as the between-subjects factors found significant main effects of fluid [Figure 2A,B; *F*(1, 20) = 80.48, *p* < 0.0001] and day [*F*(4.959, 99.19) = 5.235, *p* = 0.0003], driven by more WAT consumed than EtOH, and progressive increases in consumption over days. To further evaluate whether changes in drinking over days were meaningful in females, we next evaluated each fluid type separately. For our female WAT group (Figure 2A), the results of our two-way RM ANOVA with day as the within-subjects factor and genotype as the between-subjects factor found a significant main effect of day [Figure 2A; *F*(4.611, 46.11) = 3.145, *p* = 0.0182]. Follow-up linear regression analyses were then performed to determine whether there were differences in the rate of escalation of consumption over days. Results confirmed statistically significant escalation of WAT consumption in both genotypes [Figure 2A; female nTg *F*(1, 118) = 14.84, *r*^2^ = 0.117, *p* = 0.0002; female P301S *F*(1, 118) = 8.629, *r*^2^ = 0.06815, *p* = 0.0040], but found no differences in slopes between genotypes (*p* = 0.4353). Identical analyses as above were then performed on our female EtOH group. The results of the two-way RM ANOVA with day as the within-subjects factor and genotype as the between-subjects factor found a significant main effect of day only [Figure 2B; *F*(3.51, 33.51) = 5.911, *p* = 0.0017]. Similar to water results, regression analysis confirmed statistically significant escalation of EtOH consumption [nTg *F*(1, 118) = 12.44, *r*^2^ = 0.09540, *p* = 0.0006; P301S *F*(1, 118) = 9.132, *r*^2^ = 0.007183, *p* = 0.0031], but no differences in the slopes between genotypes [*F*(1, 236) = 0.005741, *p* = 0.9397]. Thus, females of both genotypes increased both WAT and EtOH drinking similarly over 20 days.

For males, the results of our three-way RM ANOVA with day as the within subjects factor, and fluid and genotype as the between subjects factors found a significant main effect of fluid only [Figure 2C,D; *F*(1,20) = 42.06, *p* < 0.0001], driven by more WAT consumed vs. EtOH. To be consistent with female analyses, we then further evaluated changes in drinking over days by assessing each fluid type separately. The results of our two-way RM ANOVA with day as the within subjects factor and genotype as the between subjects factor found no significant interactions or significant main effects in WAT-consuming males (Figure 2C). Follow-up linear regression confirmed that neither genotype escalated consumption over days (male nTg *p* = 0.8654; male P301S *p* = 0.0620). For our male EtOH group, the results of our two-way RM ANOVA with day as the within subjects factor and genotype as the between subjects factor found a significant main effect of day only [Figure 2D; *F*(3.338, 33.38) = 4.117, *p* = 0.0114]. Follow-up linear regression analyses confirmed that this was due to increases in EtOH consumption over days in both nTg [*F*(1,118) = 6.191, *r*^2^ = 0.04985, *p* = 0.0142] and P301S males [*F*(1,118) = 11.95, *r*^2^ = 0.09198, *p* = 0.0008]; there were no difference in slopes between genotypes (*p* = 0.5323). Thus, males of both genotypes increased EtOH consumption, but not WAT consumption, over 20 days.

We next evaluated average fluid consumed on Days 1 vs. 20 using a three-way RM ANOVA with day as the within-subjects factor, and fluid and genotype as the between-subjects factors. For mean consumption (mL/kg) from Days 1 to 20 (Figure 2E–H) we found significant main effects of days [*F*(1,44) = 9.026, *p* = 0.0044] and fluid [*F*(1, 44) = 70.5, *p* < 0.0001]; driven by higher mean consumption on Day 20 and by higher mean consumption in the WAT-consuming (Figure 2E,F) vs. the EtOH-consuming groups (Figure 2G,H). Post hoc tests confirmed that there were no between-group differences within sex, genotype, or fluid for mean fluid consumption (mL/kg) from Days 1 to 20.

To more fully appreciate changes in motivation to consume each fluid over time, we next evaluated the development of frontloading by assessing the fraction of fluid consumed within the first 15 min over the total fluid consumed over the 2-h DID via a three-way RM ANOVA with day as the within-subjects factor, and fluid and genotype as the between-subjects factors. For fraction frontloading from Days 1 to 20 (Figure 2I–L) we found significant main effects of days [*F*(1,44) = 18.76, *p* < 0.0001], and a day*fluid interaction [*F*(1, 44) = 15.67, *p* = 0.0003]; driven by higher frontloading on Day 20 within EtOH-consuming mice (Figure 2K,L). A Tukey’s post hoc test confirmed a significant increase in frontloading from Days 1 to 20 in P301S mice (*p* < 0.0001) (Figure 2L), but not in nTg mice (*p* < 0.0754) (Figure 2K).

We next assessed recorded BEC (Figure 2M) using a two-way ANOVA and found no significant main effects or interactions. In contrast, the same analysis applied to average daily predicted BECs (see Figure S1; Figure 2N) revealed a significant main effect of sex [*F*(1,20) = 4.563, *p* = 0.0452], driven by higher EtOH consumption in females.

### EtOH induces transient hyperactivity with nTg male mice

**FIGURE 3:**
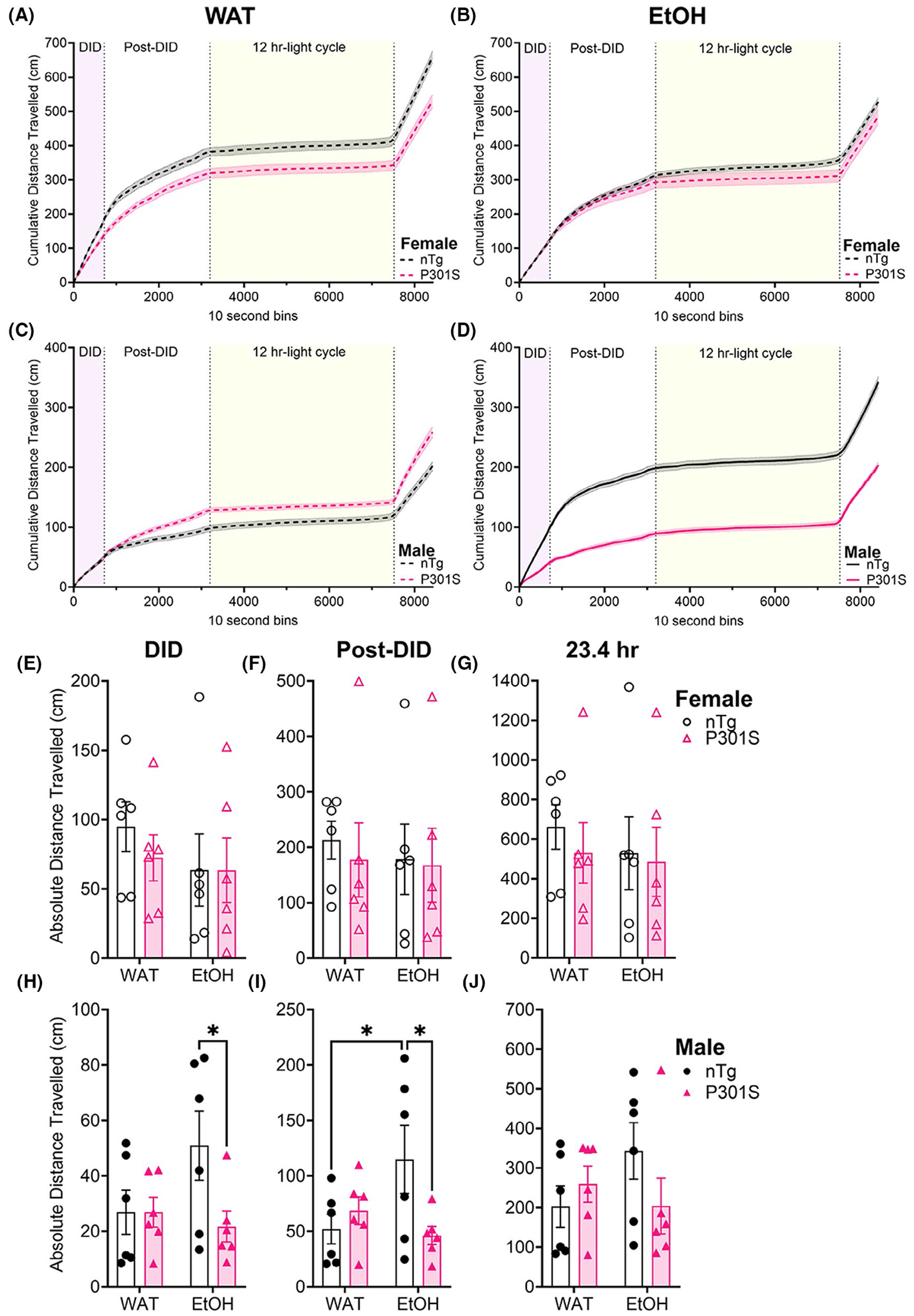
EtOH and genotype have differential impact on home cage locomotor activity in female and male mice. Cumulative distance traveled was plotted for all Female WAT (A) and EtOH (B) consuming mice as well as for all Male WAT (B) and EtOH (D) mice over 23.4 h (8424–10 s time bins). Two epochs corresponding to the absolute distance traveled during 2 h DID (purple box in A–D) and the 7 h Post-DID (white space, A–D) and the entire 23.4-h (h) time period analyzed for females (E–G) and males (H–J) separately. Bar charts in E–J illustrate the 20-day average from *n* = 6 for the absolute (or cumulative) distance traveled during that time epoch: DID (E, H), Post-DID (F, I), and the entire 23.4 experiment (G, J). During DID and during the Post-DID period, a two-way ANOVA with an uncorrected Fisher’s LSD found a significant difference in male nTg EtOH vs. P301S EtOH *p = 0.0230 (H); During Post-DID, a two-way ANOVA found a fluid*genotype interaction accounting for 18.89% total variation (*p* = 0.0307), and with an uncorrected Fisher’s LSD, a significant difference in EtOH nTg v. EtOH P301S (*p* = 0.0156) and a significant difference in WAT nTg vs. EtOH nTg (*p* = 0.0249) (I).

Home cage locomotor activity (LMA) during DID can be seen in Figure 3, where two main observations emerged (Figure 3A–D): (1) female mice have higher overall LMA compared with males (noted by scale bar differences between Figure 3A–D), and (2) EtOH consumption impacted LMA in male nTg mice (Figure 3D). To investigate further, we analyzed specific epochs of interest (2-h DID period, post-DID dark cycle period, and 23.4-h experimental day). In females, a two-way ANOVA with genotype and fluid as between-subjects factors revealed no significant main effects or interactions for LMA during any epoch (Figure 3E–G). In males, no significant main effects emerged during the 2-h DID, but a post hoc analysis using an uncorrected Fisher’s LSD revealed that nTg males consuming EtOH were significantly more active than their P301S counterparts (*p* = 0.0230) (Figure 3H). Additionally, a significant genotype*fluid interaction was observed in the post-DID period [Figure 3I, *F*(1,20) = 5.406, *p* = 0.0307]. Despite these findings, the effect did not persist over the full 23.4-h recording period [Figure 3J, *F*(1,20) = 2.498, *p* = 0.1227]. These results suggest that EtOH consumption induces transient hyperactivity in nTg male mice. The absence of significant effects across the entire experimental day may be due to high intra-group variability in LMA measurements, potentially obscuring more subtle or short-lived differences in activity levels.

### EtOH disrupts sleep and circadian rhythms in male P301S mice

**FIGURE 4:**
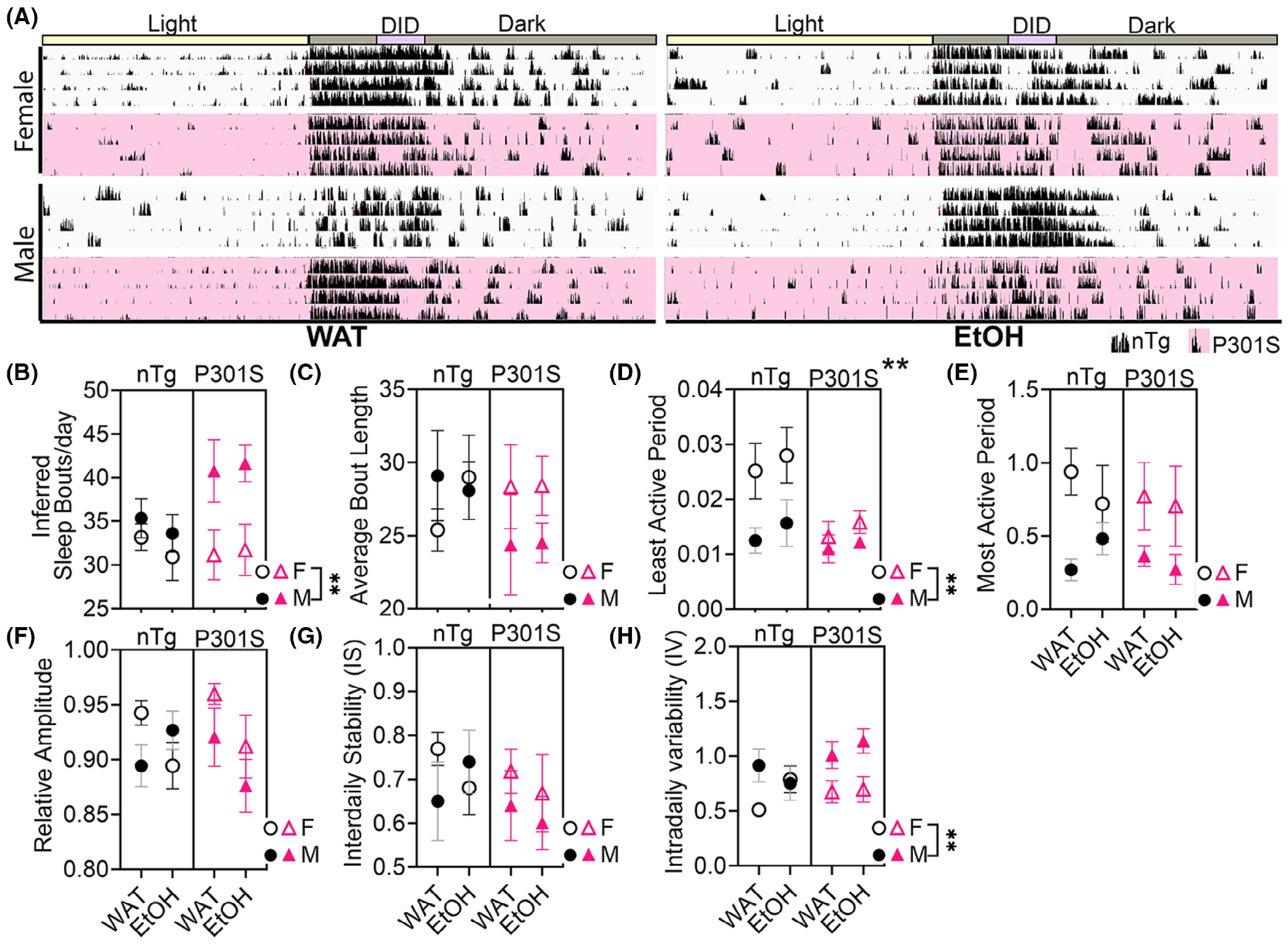
EtOH consumption leads to fragmented sleep and circadian rhythm variability in male P301S mice. (A) Actograms representing active periods (black bars) and inactive periods (absence of black bars) were plotted for the 23.4-h circadian cycle within nTg (white/black) and P301S (pink/black) female and male mice for Days 4–8, indicating the day period (light cycle, yellow) and the night period (dark cycle, black), as well as the DID period (purple). (B) There was a main effect of sex on number of inferred sleep bouts/day with male nTg and P301S mice having more inferred sleep bouts per day, even though the average bout length did not change (C). There was a main effect of genotype and sex, such that female nTg mice were more active during the least active period than female P301S mice, but this was not observed in male nTg or P301S mice (D). There were no changes in the average activity during the most active periods on sex, genotype of fluid (E). Using a three-way ANOVA did not reveal any significant differences within sex, fluid, or genotype on relative amplitude, but when the data were separated by sex there was an interaction of genotype × fluid using a two-way ANOVA (F). Interdaily stability (IS) was not impacted by fluid, sex or genotype (G). But intradaily variability (IV) was impacted by sex, such that male mice had more invariable circadian rhythm (closer to a value of 2) (H). **p ≤ 0.01.

The results of circadian rhythm analyses can be seen in Figure 4. Home cage LMA data were imported into ClockLab to generate actograms. Representative days are shown in Figure 4A, where each row represents days (e.g., D4-D8), columns reflect time in 10-s bins, and line length corresponds to activity level (i.e., longer lines indicate more activity). A three-way ANOVA with fluid, sex, and genotype as between-subjects factors identified a significant main effect of sex on the number of (inferred) sleep bouts per day [*F*(1,40) = 10.94, *p* = 0.0018], and a marginal genotype*sex interaction [*F*(1,40) = 3.896, *p* = 0.0516] (Figure 4B). Follow-up two-way ANOVAs within each sex showed a main effect of genotype in males (Figure 4B) [*F*(1,20) = 6.676, *p* = 0.0177]; with post hoc testing (uncorrected fisher’s LSD) indicating that P301S consuming EtOH had significantly more inferred sleep bouts than nTg males consuming EtOH (*p* = 0.0413). While a two-way ANOVA in female mice did not identify any main effects in (inferred) sleep bouts. Since average sleep bout length remained unchanged as a factor of genotype, sex, or fluid (Figure 4C), these data suggest that male P301S mice experience more fragmented periods of sleep compared with females (Figure 4C).

Analysis of average activity during the least active period via three-way ANOVA showed significant main effects of genotype [*F*(1,40) = 9.062, *p* = 0.0045] and sex [*F*(1,40) = 10.13, *p* = 0.0028], along with a marginal genotype*sex interaction [*F*(1,40) = 3.913, *p* = 0.0548] (Figure 4D). In males, no significant effects emerged. However, in females, a two-way ANOVA identified a significant main effect of genotype [*F*(1,20) = 9.248, *p* = 0.0064], with post hoc analysis (uncorrected Fisher’s LSD) revealing that P301S females—regardless of fluid—were less active than nTg females. A three-way ANOVA identified a significant main effect of sex on average activity during the most active period [Figure 4E; *F*(1,40) = 12.05, *p* = 0.0013], but no further significant effects were found when data were separated by sex. These data recapitulate data observed in LMA where females were more active than males (Figure 3).

Relative amplitude (RA), a measure of circadian rhythm consistency occurring over the 20 days, was not impacted by genotype, sex, or fluid when assessed in a three-way ANOVA. However, sex-specific two-way ANOVAs revealed a significant main effect of fluid in females [(Figure 4F), *F*(1,20) = 6.229, *p* = 0.0214], with EtOH reducing RA in both nTg and P301S mice. No effects were found in males [(Figure 4F), *F*(1,20) = 0.07208, *p* = 0.7911], suggesting that females may be more susceptible to EtOH-induced circadian disruption.

To further evaluate circadian rhythm integrity, we examined IS and IV to assess their day-to-day consistency and their daily rhythmicity of circadian behavior, respectively. IS was unaffected by any factor (Figure 4G), but IV showed a significant main effect of sex [Figure 4H *F*(1,40) = 11.52, *p* = 0.0016]. A two-way ANOVA within genotype groups revealed that this was driven by increased IV within male P301S mice, regardless of fluid [*F*(1,20) = 11.83, *p* = 0.0026], indicating more fragmented circadian patterns, which is consistent with the observation of fragmented sleep in male P301S mice.

### EtOH induces trait abnormalities within P301S mice

**FIGURE 5:**
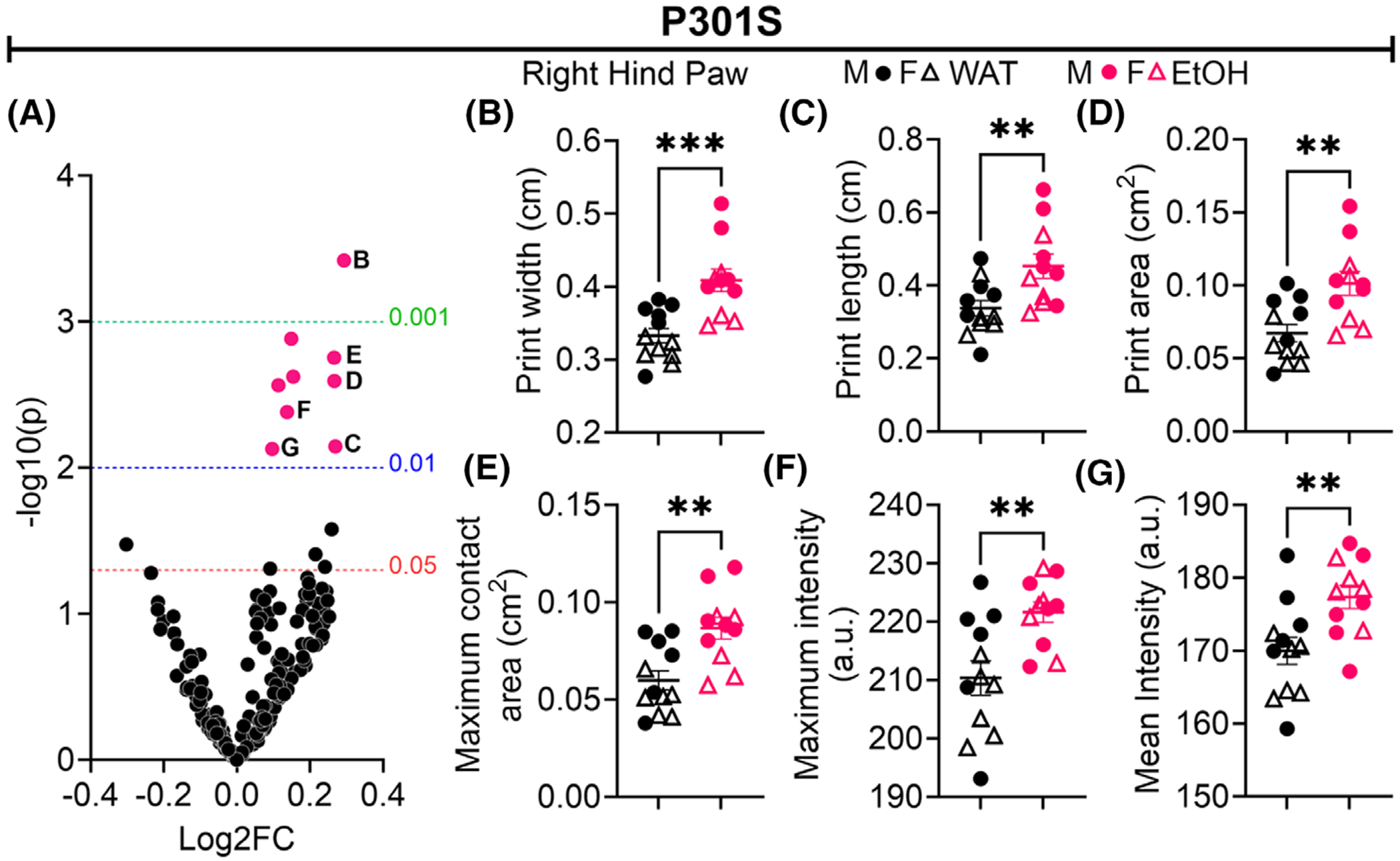
EtOH significantly alters gait in P301S mice consuming alcohol (EtOH) compared with water (WAT). Data obtained from the Catwalk XT^®^ (v. 5.8) on the second run of mice following a 21 DID procedure was normalized, Z-scored, and compared using a false discovery rate (FDR)-corrections approach for multiple comparisons and plotted as a volcano plot (A). Raw values from the CATWALK that survived the negative log10 *p* value of <0.01, which corresponded to all Right Hind (RH) Paw Traits, are plotted in (B) RH Mean Print Width, (C) RH Print Length, (D) RH Print Area, (E) RH Maximum Contact Area, (F) RH Maximum Intensity, (G) RH Mean Intensity. Unlabeled dots in (A): RH Mean Intensity of the 15 most intense pixels, RH Max Contact Max Intensity Mean, RH Max Contact Mean Intensity Mean. *N* = 11–12/group, 6 M (closed circles) 5F (open triangles), Student’s *t*-test, **p ≤ 0.01, ***p ≤ 0.001. nTg mice are assessed in Figure S2.

The results of gait assessments acquired by the Catwalk XT^®^ test can be seen in Figure 5. Since gait impairments are typically not observed in P301S mice until closer to 4 months of age, we did not observe, nor anticipate any differences in the WAT-nTg compared with the WAT-P301S (Sun et al., 2020). EtOH consumption induced alterations only in the P301S mice, and notably, all significant changes were restricted to traits associated with the right hind paw (Figure 5A) that met the FDR-corrected cutoff of *p* < 0.01: print width (Figure 5B) (student’s *t*-test, *p* = 0.0004), print length (Figure 5C) (student’s *t*-test, *p* = 0.0071), print area (Figure 5D) (student’s *t*-test, *p* = 0.0025), maximum contact area (Figure 5E) (student’s *t*-test, *p* = 0.0018), maximum intensity (Figure 5F) (student’s *t*-test, *p* = 0.0041), and mean intensity (Figure 5G) (student’s *t*-test, *p* = 0.0074) were all significantly increased within P301S mice as a consequence of EtOH. No such gait abnormalities were observed in the EtOH nTg mice compared with the WAT-consuming nTg mice; data are plotted using the same volcano plot analysis in (Figure S2).

### EtOH consumption enhances pathological tau accumulation

**FIGURE 6:**
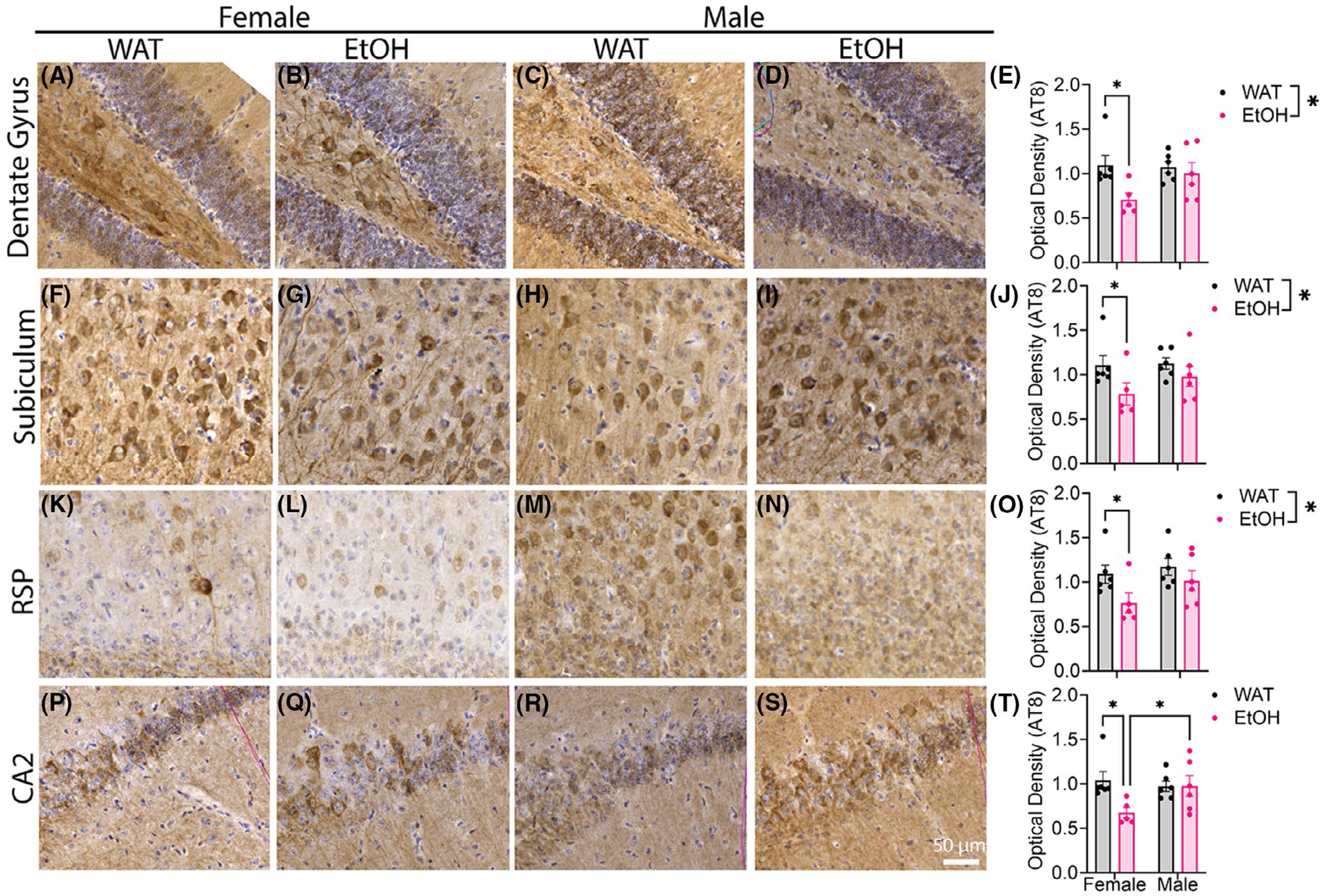
Alcohol significantly decreases the optical density (OD) of AT8 within DG, SUB, and RSC and CA2 of P301S consuming EtOH. AT8 immunoreactivity was measured within the dentate gyrus of Female WAT (A), Female EtOH (B), Male WAT (C), and Male EtOH (D) P301S mice and was (E) significantly decreased as a main effect of fluid, but a post hoc test found this was mediated by differences within females. AT8 immunoreactivity was also measured within the SUB of Female WAT (F), Female EtOH (G), Male WAT (H), and Male EtOH (I), and was also found to be decreased in the same direction (J). AT8 immunoreactivity was also measured within the RSC of the Female WAT (K), Female EtOH (L), Male WAT (M), and Male EtOH (N), and was also found to be decreased in the same direction (O). AT8 immunoreactivity was also measured within the CA2 of the Female WAT (P), Female EtOH (Q), Male WAT (R), and Male EtOH (S), and was also found to be decreased in the same direction (T). *N* = 5–6/group two-way ANOVA with a main effect of fluid, *p ≤ 0.05 (E, J, O). Multiple comparisons conducted with an uncorrected Fisher’s LSD, Female WAT vs. Female EtOH *p ≤ 0.05 (E, J, O, T), and an uncorrected Fisher’s LSD, Male EtOH vs. Female EtOH *p ≤ 0.05 (T). The percent immunoreactivity of AT8 within the CA2 is assessed within Figure S3.

Results of AT8 immunoreactivity in P301S mouse brains are shown in Figure 6. Within female P301S mice, EtOH exposure reduced AT8 OD in several regions: the dentate gyrus (DG, Figure 6A–E) [main effect of fluid, *F*(1,19) = 5.475, *p* = 0.0304; uncorrected Fisher’s LSD, Female WAT vs. EtOH, *p* = 0.0135], the Subiculum (SUB, Figure 6F–J) [main effect of fluid, *F*(1,19) = 5.027, *p* = 0.0371; Uncorrected Fisher’s LSD, Female WAT vs. EtOH, *p* = 0.0451], the Retrosplenial Cortex (RSC, Figure 6K–O) [main effect of fluid, *F*(1,19) = 5.106, *p* = 0.0358; Uncorrected Fisher’s LSD, Female WAT vs. EtOH, *p* = 0.0487], and the CA2 [Uncorrected Fisher’s LSD, Female WAT vs. EtOH, *p* = 0.0108; EtOH Female vs. EtOH Male, *p* = 0.0312]. These results suggest that EtOH reduces OD across these regions, most significantly within female P301S mice. However, the percent AT8 immunoreactivity (across all intensity bins) was greatest within the CA2 in EtOH-consuming P301S compared with WAT-consuming P301S mice driven by differences in male P301S mice (Figure S3) {Average IR (A) [main effect of fluid, *F*(1,19) = 6.909, *p* = 0.0165; Uncorrected Fisher’s LSD, Male WAT vs. EtOH, *p* = 0.0338]; Weak IR (B) [main effect of fluid *F*(1,19) = 7.079, *p* = 0.0154; Uncorrected Fisher’s LSD, Male WAT vs. EtOH, *p* = 0.0436]; Moderate IR (C) [main effect of fluid *F*(1,19) = 4.910, *p* = 0.0391, Uncorrected Fisher’s LSD, Male WAT vs. EtOH, *p* = 0.0218]; Strong IR (D) [main effect of fluid *F*(1,19) = 5.066, *p* = 0.0364, Uncorrected Fisher’s LSD, Male WAT vs. EtOH, *p* = 0.0364]}. Together, these findings suggest that EtOH could be promoting the spread of pTau—potentially driving clearance or reduced accumulation within DG, SUB, and RSC, enhancing pTau deposition within downstream targets like the CA2.

**FIGURE 7:**
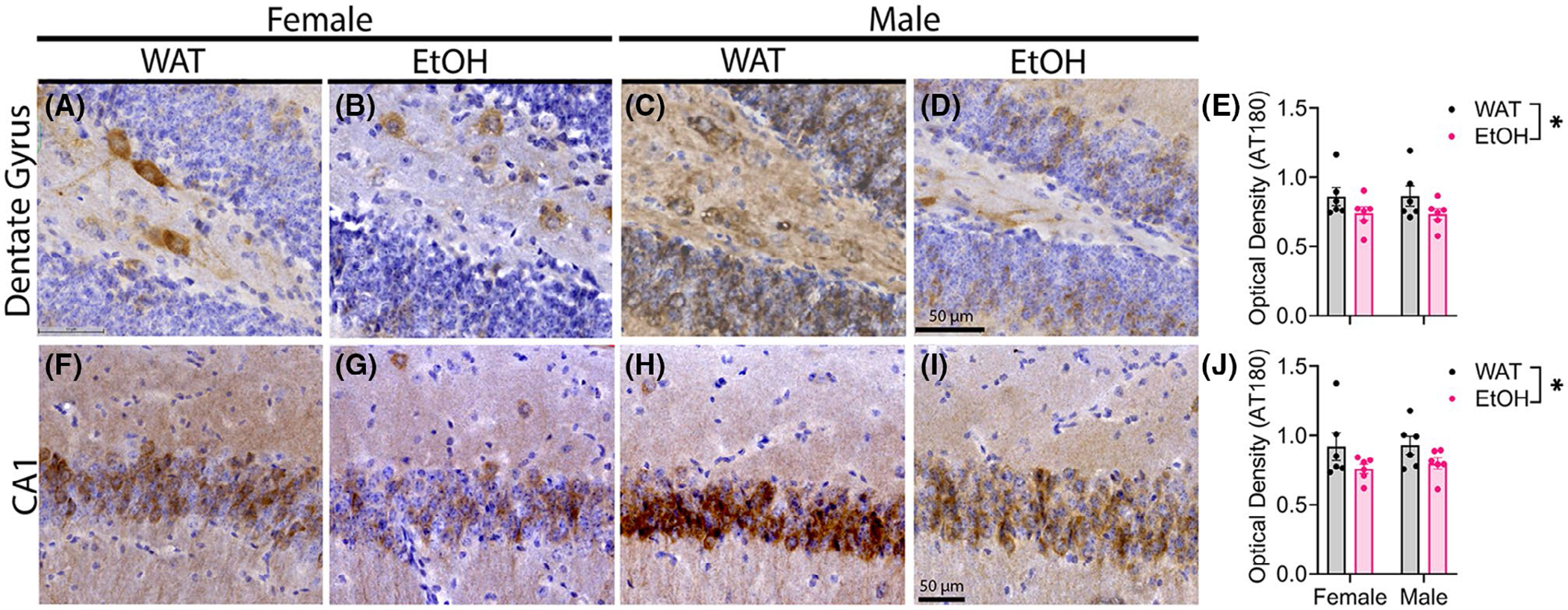
Alcohol significantly decreases the optical density of pTau (AT180) within the CA1 and DG of P301S mice. AT180 immunoreactivity within the DG of Female WAT (A), Female EtOH (B), Male WAT (C), and Male EtOH (D) was significantly reduced as a main effect of EtOH (E). AT180 immunoreactivity within the CA1 of Female WAT (F), Female EtOH (G), Male WAT (H), and Male EtOH (I) was significantly reduced as a main effect of EtOH (J) *N* = 6/group; two-way ANOVA with a main effect of fluid, *p ≤ 0.05.

Results of AT180 immunoreactivity within the brain regions of the P301S mice are shown in Figure 7, which displayed a similar laminar pattern to AT8. EtOH exposure significantly decreased the OD of AT180 within the CA1 (Figure 7A–E) [main effect of fluid, *F*(1,20) = 4.831, *p* = 0.0399] and the DG (Figure 7F–J) [main effect of fluid, *F*(1,20) = 4.677, *p* = 0.0429]. These findings suggest that the more stable nature of phosphorylation recognized by AT180 may be less susceptible to EtOH-induced modulation compared with the transient pTau species recognized by AT8.

### EtOH promotes microglia phenotype switching in female P301S mice

**FIGURE 8:**
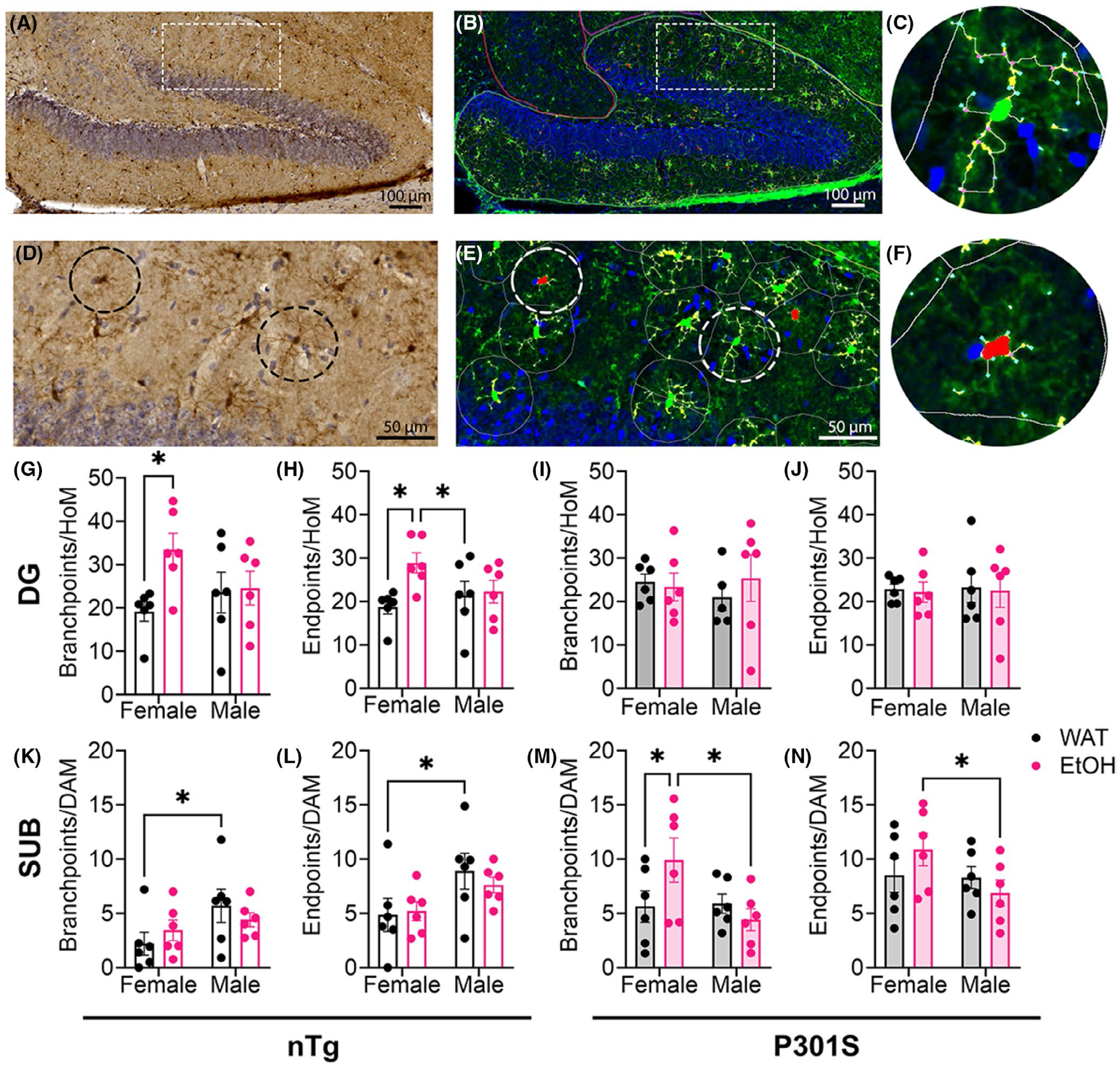
EtOH significantly increases the number of branch points and end points within homeostatic microglia of the dentate gyrus (DG) in nTg mice following EtOH exposure. (A) The region of interest drawn in the DG of a nTg WAT-consuming male mouse. (B) iba1 Diaminobenzidine IHC has been deconvolved using HALO^®^ to assess microglia morphology. (C) Magnified image of rectangular box in (A). (D) Magnified image of rectangular box in (B), which illustrates two separate populations of microglia assessed, homeostatic microglia (HoM, green, E), and damage-associated microglia (DAM, red, F). Pink dots represent branchpoints and cyan dots represent Endpoints within (E) and (F). EtOH significantly increased the number of branch points (G) and end points (H) within HoM only in female nTg mice in the DG, but did not impact the branchpoints (I) or Endpoints (J) within the HoM of the DG in P301S mice. There were no changes within the DAMs of the DG (assessed in Figure S4A–D). Interestingly, there were no impacts in the branchpoints (K) or the endpoints (L) of DAM within the subiculum (SUB) as a function of fluid in nTg mice, but female nTg mice had fewer endpoints as a function of sex in the WAT group (L). Interestingly, EtOH-consuming female P301S mice had significantly higher branchpoints (M) and endpoints (N) within the DAMs of the SUB compared with other groups analyzed. There were no changes within the HoMs of the SUB (assessed in Figure S4E–H). *N* = 6/group two-way ANOVA, with a Tukey’s post hoc test *p < 0.05.

Results of Iba1 immunoreactivity within the DG are shown in Figure 8. First, iba1-stained sections were scanned in at a 20x magnification (Figure 8A) and were processed through a deconvolution algorithm (Figure 8B). Magnified images are shown of both the original scans (Figure 8C) and the deconvolved image (Figure 8D). To conduct these experiments, we used HALO^®^ imaging software and chose a threshold of 1.5 for the “activation” score, to quantify the total number of microglia and to differentiate the morphological phenotype of microglia, specifically the differences between homeostatic microglia (HoM; Figure 8E) and damage-associated microglia (DAM; Figure 8F) across eight different brain regions. To assess the impact of EtOH on neuroinflammation using microglia phenotype, we performed separate two-way ANOVAs within each genotype (nTg and P301S), using sex and fluid as between-subjects factors. This approach allowed us to evaluate region and sex-specific effects on EtOH in alignment with previous pTau results. We found significant changes within the DG and SUB. EtOH significantly increased branch points (Figure 8G) [main effect of fluid *F*(1, 20) = 4.193, *p* = 0.0540] and endpoints (Figure 8H) [main effect of fluid *F*(1, 20) = 4.784, *p* = 0.0408] within HoMs, driven by changes in nTg female mice [Uncorrected Fisher’s LSD, Branchpoints: *p* = 0.0138; Endpoints: *p* = 0.0101], but there were no significant differences within branch points (Figure 8I) or endpoints (Figure 8J) of HoMs within the P301S mice. These data suggest that EtOH enhances HoMs within the DG of nTg females, possibly reflecting adaptive or anti-inflammatory responses.

When assessing the DAMs of the DG, we did not observe any significant differences (Figure S4A–D). Within the SUB, there were no significant differences within branchpoints of DAMs within the nTg mice (Figure 8K), but there seemed to be a marginally significant reduction in endpoints of DAMs driven by a main effect of sex (Figure 8L) [*F*(1,20) = 4.142, *p* = 0.0542; Uncorrected Fisher’s LSD, nTg Male WAT vs. nTg Female WAT, *p* = 0.0352]. EtOH significantly increased the branchpoints (Figure 8M) [Uncorrected Fisher’s LSD, Male EtOH vs. Female EtOH, *p* = 0.0129; Female WAT vs. Female EtOH, *p* = 0.0467] and endpoints within the DAMs of female mice consuming EtOH (Figure 8N) [Uncorrected Fisher’s LSD, Male EtOH vs. Female EtOH, *p* = 0.0481]. When assessing the HoMs within the SUB, we did not observe any significant differences (Figure S4E–H). These data suggest that EtOH induces morphological activation of DAMs within the SUB, specifically in female P301S, without the same changes in their HoM counterparts.

## DISCUSSION

Using the DID experimental paradigm in a tauopathy model (P301S), this study found that 3 weeks of moderate alcohol consumption accelerated gait impairments and altered pTau density and damage-associated microglia. Other notable effects were either fluid-, sex-, or genotype-specific. These results support reports that alcohol exposure alters pTau, resulting in altered or exacerbated neurobehavioral outcomes.

Both genotypes of mice engaged in moderate alcohol consumption, with P301S and nTg littermates achieving similar BECs (Figure 2N; 41.1 ± 7.1 mg/dL collapsed on genotype/sex). Although these levels, on average, did not reach the binge drinking threshold the DID paradigm was originally designed to achieve in C57BL/6J mice (≥80 mg/dL), they were indeed pharmacologically relevant. The only other study to evaluate alcohol drinking levels in P301S mice found higher total ethanol consumption and preference in P301S males compared with WT males over 16 weeks of intermittent 24-h alcohol access (Downs et al., 2022). While our study did not find genotype differences in mean EtOH consumption using a 3-week exposure, it remains possible that longer access using DID may have revealed similar effects. Another important observation was the development of alcohol frontloading in just the P301S mice (Figure 2K,L)—a phenomenon thought to reflect increases in the motivation to experience the subjective effects of alcohol (Ardinger et al., 2022). Despite this evidence of increased motivation for alcohol, our findings suggest drinking levels were influenced by generally lower voluntary alcohol drinking in C3H inbred substrains compared with inbred C57BL/6J mice (Kahn, 1975), potentially due to innate differences in sensitivity to alcohol’s aversive properties. For instance, C3H mice exhibited a nearly 100% conditioned taste aversion (CTA) to alcohol, while C57BL/6J mice showed almost none (Moore et al., 2013). It is difficult to compare our findings to the small existing literature due to meaningful differences in alcohol exposure methods between studies, which range from nonvoluntary intragastric alcohol administration (Barnett et al., 2022; Tucker et al., 2022) to voluntary 24-h two-bottle choice assays (Day et al., 2023; Downs et al., 2022). Nonetheless, this is the first study to examine voluntary alcohol consumption using DID in this specific pTau hybrid mouse line, which we found demonstrates reliable and meaningful voluntary alcohol consumption suitable for investigating alcohol*AD interactions.

Hyperactivity is a common phenotype observed within mouse models of tauopathy (Takeuchi et al., 2011), particularly in male P301S mice (Dumont et al., 2011). Thus, it was initially surprising that our home cage LMA data showed no genotype difference in WAT-consuming mice of either sex, but significant hyperactivity in EtOH-consuming male nTg mice only (Figure 3E–J). The initial report of P301S hyperactivity was based on comparisons to a C57BL/6J back-crossed congenic line (Takeuchi et al., 2011), and not the C57BL/6J x C3H/HeJ F1 hybrid littermates (JAX strain ID #100010). Differences in locomotion between studies may also stem from testing in an open field versus a home cage. Regardless, the differences observed in EtOH-consuming male nTg mice were greater than in WAT-consuming male nTg mice, suggesting that alcohol may be directly responsible for the hyperactivity, and that alcohol-induced locomotor stimulation scales with exposure. However, neither the distance traveled during DID [*F*(1,4) = 0.3930, *r*^2^ = 0.08946, *p* = 0.5647] nor the distance traveled during the Post-DID interval [*F*(1,4) = 0.7634, *r*^2^ = 0.1603, *p* = 0.4316] were significantly correlated with EtOH consumption within male nTg mice. While the lack of an association between locomotion and alcohol consumption does not rule out a direct role for alcohol in increasing ambulation, it does suggest that male nTg mice may be more behaviorally reactive to experimental manipulations (i.e., noise/sipper manipulations) compared with other groups. This is the first study to examine genotype*sex alterations in home cage LMA in the P301S line, which ultimately found no meaningful interactions between pTau and alcohol.

Circadian rhythm is a prominent daily behavioral and physiological cycle that enables organisms to respond to and anticipate changes in their environment (Ruan et al., 2021). Notably, circadian rhythm can become disrupted in both people and mice as a function of alcohol use (Meyrel et al., 2020), and such disruptions are often moderated by age (Ruby et al., 2017) and sex (de Zavalia et al., 2023). In AD, circadian rhythm is commonly dysregulated and can manifest as “sundowning”—a phenomenon where patients experience increased agitation during either early morning or evening hours (Volicer et al., 2001). AD patients also frequently suffer from sleep disruptions, which can further accelerate disease progression (Musiek et al., 2015). Similarly, P301S mice exhibit fragmented sleep, reflecting early disruptions to circadian behavior (Han et al., 2022). To determine whether alcohol impacts circadian behavior in a genotype-specific manner, we generated and analyzed actograms using LMA (Figure 4A). Of the various circadian parameters evaluated, only (inferred) sleep bouts (Figure 4B), least active periods (Figure 4D) and intradaily variability (IV; Figure 4H) were significantly affected by one or more of our variables of interest. First, we observed a significant increase in the number of (inferred) sleep bouts in male P301S mice (Figure 4B), without a corresponding increase in (inferred) sleep bout length (Figure 4C). This suggests that pTau accumulation may contribute to sleep fragmentation—reflected as shorter, more frequent periods of inactivity—but only in male P301S. These findings are in agreement with prior work demonstrating sleep deficits in P301S mice as early as 3 months of age, which were associated with accelerated tau pathology and cognitive decline (Martin et al., 2024). However, that work did not assess the influence of EtOH consumption. To complement our analysis of inferred sleep behavior, we employed nonparametric circadian rhythm metrics (Gonçalves et al., 2015), including assessments of activity during the least (Figure 4D) and most active periods (Figure 4E), RA (Figure 4F), IS (Figure 4G) and IV (Figure 4H). Activity during the least active period showed clear sex-specific effects, with female nTg mice exhibiting higher baseline activity than males—consistent with our prior LMA findings (Figure 3). Interestingly, this sex difference was not observed in P301S mice, suggesting that tau pathology may suppress the typically higher activity level observed in females. These results are also reflected in our catwalk gait analysis, where both male and female P301S mice exhibited hind paw impairments exacerbated by EtOH (Figure 5A). While our current experiments were not designed to assess endogenous circadian rhythms—an approach that would require testing in the absence of environmental cues such a light (Eckel-Mahan & Sassone-Corsi, 2015)—future studies should explore circadian rhythmicity. This would enable a deeper understanding of how alcohol and tau pathology may interact to influence internal circadian timing and drift.

One of the most common neurological deficits seen in individuals with an alcohol use disorder is ataxic gait (Mitoma et al., 2021), due to the vulnerability of the cerebellum to ethanol (Abdallah et al., 2021). Similarly, patients with AD and primary tauopathies often exhibit progressive motor deficits, including rigidity, bradykinesia, difficulty initiating movement and gait instability (Kurlan et al., 2000; Murley et al., 2020). These deficits often manifest as reduced walking speed and increased variability in velocity (Cohen & Verghese, 2019), which some consider an early hallmark of AD (Muňoz et al., 2010). Recent work in humans shows that blood-based biomarkers—including GFAP (neuroinflammatory marker), NfL (Neurofilament Light, neuronal atrophy), and pT181 (pTau marker)—predict age-related declines in gait velocity (Ali et al., 2025). To assess motor coordination as a function of alcohol and tauopathy, we used the Catwalk XT Assay 48 h after the final DID session. We selected this assay based on prior validation in a mouse model of prenatal ethanol exposure (Jacquez et al., 2020). Strikingly, EtOH consumption led to significant impairments in right hind paw traits exclusively in P301S mice (Figure 5A), with no comparable deficits observed in nTg controls (Figure S2). While gait abnormalities have previously been reported in P301S (Sun et al., 2020), those were not observed until 4 months of age. Here, we report for the first time that alcohol exposure accelerates gait deficits in P301S mice as early as 3 months of age. We speculate that the unilateral hind paw deficits seen in EtOH-exposed P301S mice may result from increased pTau aggregation or microglia activation in the contralateral (left) hemisphere, potentially involving the left cerebellum. However, only the right hemisphere was processed for IHC. Further investigation is warranted to determine whether asymmetric gait deficits are driven by lateralized brain pathology in the context of pTau and alcohol.

To investigate the impact of EtOH consumption on tau pathology, we probed brain tissue from P301S mice using two antibodies against hyperphosphorylated tau: AT8 (Ser202/Thr205), which marks transient pTau (Hitrec et al., 2021), and AT180 (Thr231), considered more stable and resistant to dephosphorylation as well as an incipient marker of AD in human disease (Ashton et al., 2021). We selected both antibodies since the hyperphosphorylation at both sites can determine both the spread and the morphology of pTau development (Hu et al., 2016). We found that AT8 immunoreactivity was significantly elevated in the CA2 of EtOH-consuming P301S mice, compared with WAT (Figure S3A–D). In contrast, AT8 OD was reduced within brain regions upstream of CA2—including the RSC (Figure 6O), SUB (Figure 6J), and DG (Figure 6E)—supporting our working hypothesis that alcohol may facilitate pTau spread, ultimately leading to accumulation in the CA2. This aligns with prior work suggesting that CA2 may act as a “sink” for propagating tau species (Walker et al., 2021). AT180 OD was significantly reduced in both male and female P301S mice within the CA1 (Figure 7E) and DG (Figure 7J), but unlike AT8, we did not observe the same concomitant increase in CA2 immunoreactivity. This may reflect the slower dynamics of AT180-associated pTau accumulation compared with AT8, and possible permanent pathogenic tau spread, visualized by AT180, could take a longer amount of time than the rapidly phosphorylated AT8 site. While a recent study illustrated elevated levels of AT8 within the Locus Coeruleus (LC) of older (9 months) P301S mice (Downs et al., 2025), it remains unclear whether EtOH could further exacerbate this phenotype. Overall, the AT8 results suggest that alcohol may reduce pTau OD by enhancing its spread, limiting the opportunity for aggregation and inducing increased aggregation within the CA2.

Neuroinflammation is increasingly recognized as a key contributor to both AD and alcohol use (León et al., 2022). First, neuroinflammation and pathological tau aggregation processes are tightly linked in AD (Leng & Edison, 2021), and elevated inflammatory markers have been detected in the CSF of individuals with AUD (Lékó et al., 2023). In the 3x-Tg-AD model, adolescent alcohol exposure accelerates AD pathology via proinflammatory mechanisms (Barnett et al., 2022). Based on this, we assessed neuroinflammation using the microglial marker, ionized calcium-binding adaptor molecule 1 (iba1), which allowed us to distinguish damage-associated microglia (DAM) from homeostatic microglia (HoM). Within the DG, EtOH-exposed nTg females had significantly more ramified HoM—indicated by increased branch points (Figure 8G) and endpoints (Figure 8H)—while P301S females showed no such changes (Figure 8I,J). These data suggest that alcohol could enhance basal immune surveillance in nTg females. In contrast, in the SUB, EtOH reduced DAM endpoints in nTg females (Figure 8L), indicating a more proinflammatory phenotype. Conversely, in P301S females, EtOH significantly increased branch points in the DAM (Figure 8M) suggesting a more hyper-ramified, vigilant microglia state—consistent with findings in women with AD (Casaletto et al., 2022). These data highlight emerging sex-dependent neuroimmune responses to alcohol (Cruz et al., 2023). In P301S females, hyper-vigilant DAMs in the SUB could contribute to reduced pTau OD (Figure 6F), potentially via enhanced clearance. However, such clearance may paradoxically promote pTau seeding and spreading, as reported previously (Maphis et al., 2015; Wang et al., 2022). Overall, this is the first study to characterize microglia morphology in P301S mice following voluntary alcohol exposure.

While this is still one of the first studies to use a voluntary DID model to explore the impact of EtOH consumption on pathological outcomes relevant to Alzheimer’s disease, namely tauopathy and neuroinflammation, there are several limitations to this work. First, our animals consumed a moderate amount of EtOH. Although one could argue moderate alcohol consumption is more relevant to typical patterns of use, other models are clearly needed for evaluating the impact of more excessive exposures on AD risk (Barnett et al., 2022). Since this specific tauopathy model exhibits rapidly developing pathology, mice were exposed to alcohol early and evaluated at a young age after a relatively brief (21 days) exposure period. Future work should prioritize less pathogenic models in favor of slower tau-development similar to what is observed in the humanized tau line (Maphis et al., 2015), to allow for better assessments of the longitudinal impacts of EtOH on pTau and neuroinflammation throughout the lifespan.

In conclusion, moderate alcohol use in P301S mice leads to alterations in gait which could be a function of the aggregation of pathological tau and alterations within the phenotype of damage-associated microglia within the hippocampus of P301S mice. These results contribute to the growing body of literature examining the role of alcohol consumption and pave the way for future studies exploring the mechanisms by which alcohol may interact with these and other neurobiological and behavioral alterations relevant to AD onset and pathogenesis.

## Supplementary Material

Supporting Information: Figures

Supporting Information: Table3

Supporting Information: Table2

Supporting Information: Table1

Additional supporting information can be found online in the Supporting Information section at the end of this article.

## Data Availability

The data that support the findings of this study are available from the corresponding author upon reasonable request.
