# Supplementary material for: Alcohol consumption in P301S mice accelerates gait impairments, modifies aggregation of pathological tau and alters microglia within the hippocampus
Source: Alcohol Clin Exp Res (Hoboken). Author manuscript; Available in PMC 2026 Feb 25. (PMC12934799; doi:10.1111/acer.70123)
Supplement: Supporting Information: Figures [file NIHMS2142986-supplement-Supporting_Information__Figures.zip › acer70123-sup-0002-FigureS1-S4@Supplementary Figure Legends.docx]

# Supplementary Figure Legends:

**Supplementary Figure 1: Blood Ethanol Concentration predicted using linear regression and EtOH consumption. A.** Immediately following cessation from EtOH bottles on D21 of DID, we used the ANALOX to measure the BEC from the periorbital sinus blood of each EtOH consuming animal (n=24). The slope of the data is significantly non-zero, F(1,21) = 57.50, p< 0.0001]. **B.** When the BEC data is separated by genotype (nTg, black; P301S, pink) and sex (M, male, circle; F, female, triangle), only the nTg data generates a significant slope for M and F [For male data, F(1,4) = 19.55, p = 0.0115; For female data, F(1,4) = 31.36, p=0.0050]. **C.** We used linear regression analysis to plot predicted BECs on every single day of the DID, for D1-D20 using the equation generated by the linear regression in **A**. **C.** Predicted BECs for each day for each genotype (nTg = black, and P301S = pink) and sex (M = circle and F= triangle) are plotted, with the mean value of BECs for each genotype plotted as solid lines with diamonds. 2-way RM ANOVA found a significant impact of day on the data, illustrating a significant escalation of EtOH consumption across both genotypes F(4.884, 107.4)=8.493, p< 0.0001. **D.** Splitting the average data by genotype and sex generates a 3-way ANOVA which found a main impact of day [F(19, 38)=8.503, P < 0.0001] and sex [ F(1,20) = 4.447, p = 0.0478] on the data.

**Supplementary Figure 2. EtOH does not induce disruptions in Right hind gait traits within nTg mice. A.** Gait traits of nTg have been Normalized and Z-scored and plotted as a volcano plot where multiple T-tests were conducted and none survived False discover rate (FDR)-correction above p ≤ 0.01. The raw data of the same traits disrupted by EtOH in P301S mice, have been acquired from the Catwalk XT® and plotted in **B**-**G**, where no significant differences between WAT or EtOH emerged. N=11-12/group, 6M/6F, Student’s T-test, n.s. = not significant.

**Supplementary Figure 3. EtOH significantly increases percent immunoreactivity within the CA2 of P301S mice.** AT8 immunoreactivity (IR) was evaluated within the CA2 using HALO® image processing. **A.** EtOH-consuming P301S mice, specifically males had significantly elevated level of Average Percent IR. This was seen within the (**B**) weakly stained, (**C**) moderately-stained and (**D**) strongly-stained IR of the AT8 within the CA2 of P301S mice. N = 5-6/group, 2-way ANOVA, main effect of fluid, p < 0.05, Uncorrected Fisher’s LSD, Male WAT vs. EtOH, p < 0.05.

**Supplementary Figure 4. EtOH does not significantly alter the number of branch points or end points within DAM of the DG or HoM within the SUB.** EtOH did not alter the number of branch points (**A**) or end points (**B**) within DAM of nTg mice in the DG, nor the branchpoints (**C**) or Endpoints (**D**) within the DAM of the DG in P301S mice. There were no significant differences within the branchpoints (**E**) or the Endpoints (**F**) of HoM within the SUB as a function of fluid in nTg mice, nor within the branchpoints (**G**) or endpoints (**H**) within the HoMs of the SUB in the P301S mice. N = 6/group 2-way ANOVA, with a Tukey’s Post-Hoc test * p <0.05.
