# Supplementary figures and images for: Alcohol consumption in P301S mice accelerates gait impairments, modifies aggregation of pathological tau and alters microglia within the hippocampus
Source: Alcohol Clin Exp Res (Hoboken). Author manuscript; Available in PMC 2026 Feb 25. (PMC12934799; doi:10.1111/acer.70123)

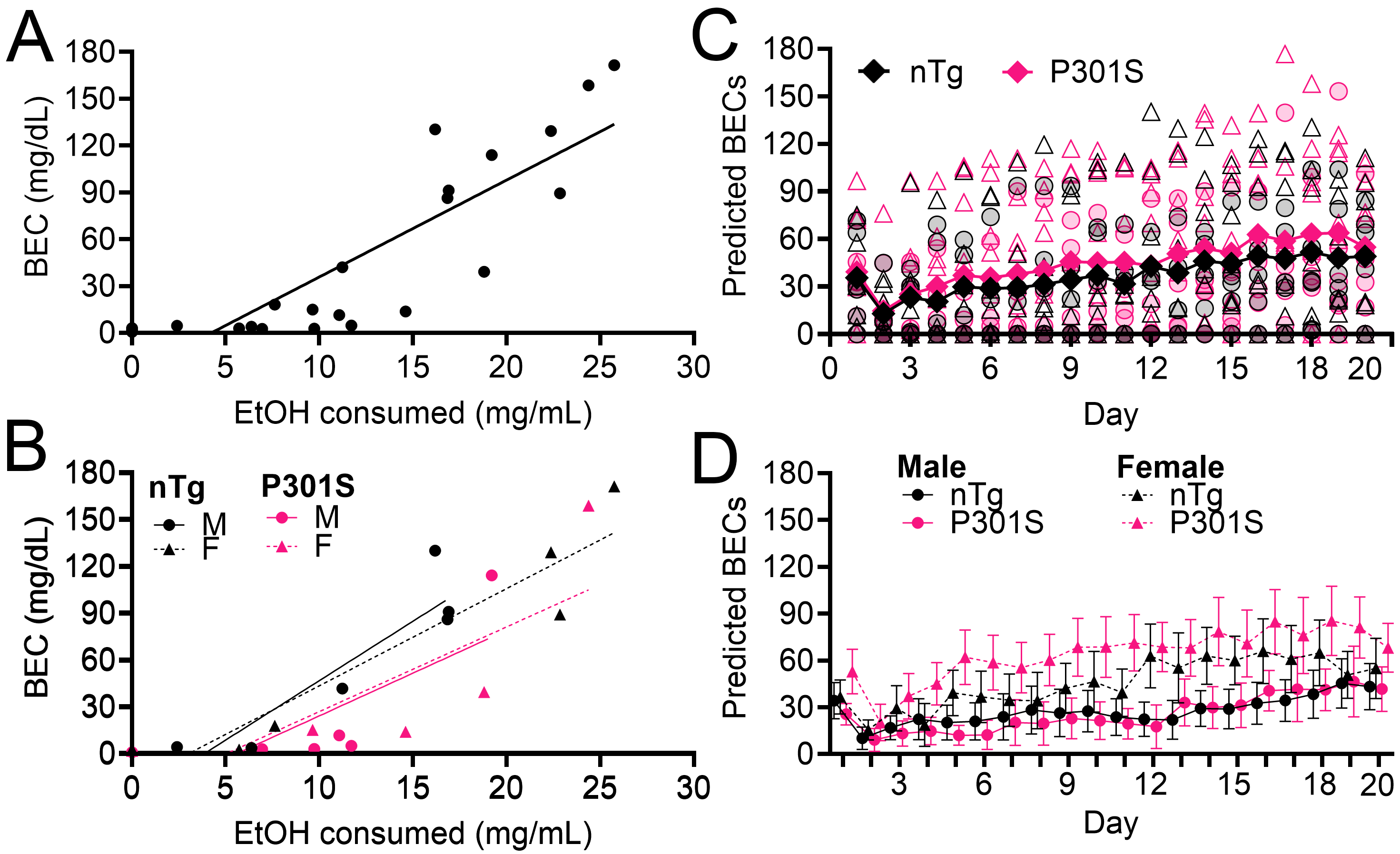

Supplement: Supporting Information: Figures [file NIHMS2142986-supplement-Supporting_Information__Figures.zip › acer70123-sup-0001-FigureS1.tif]

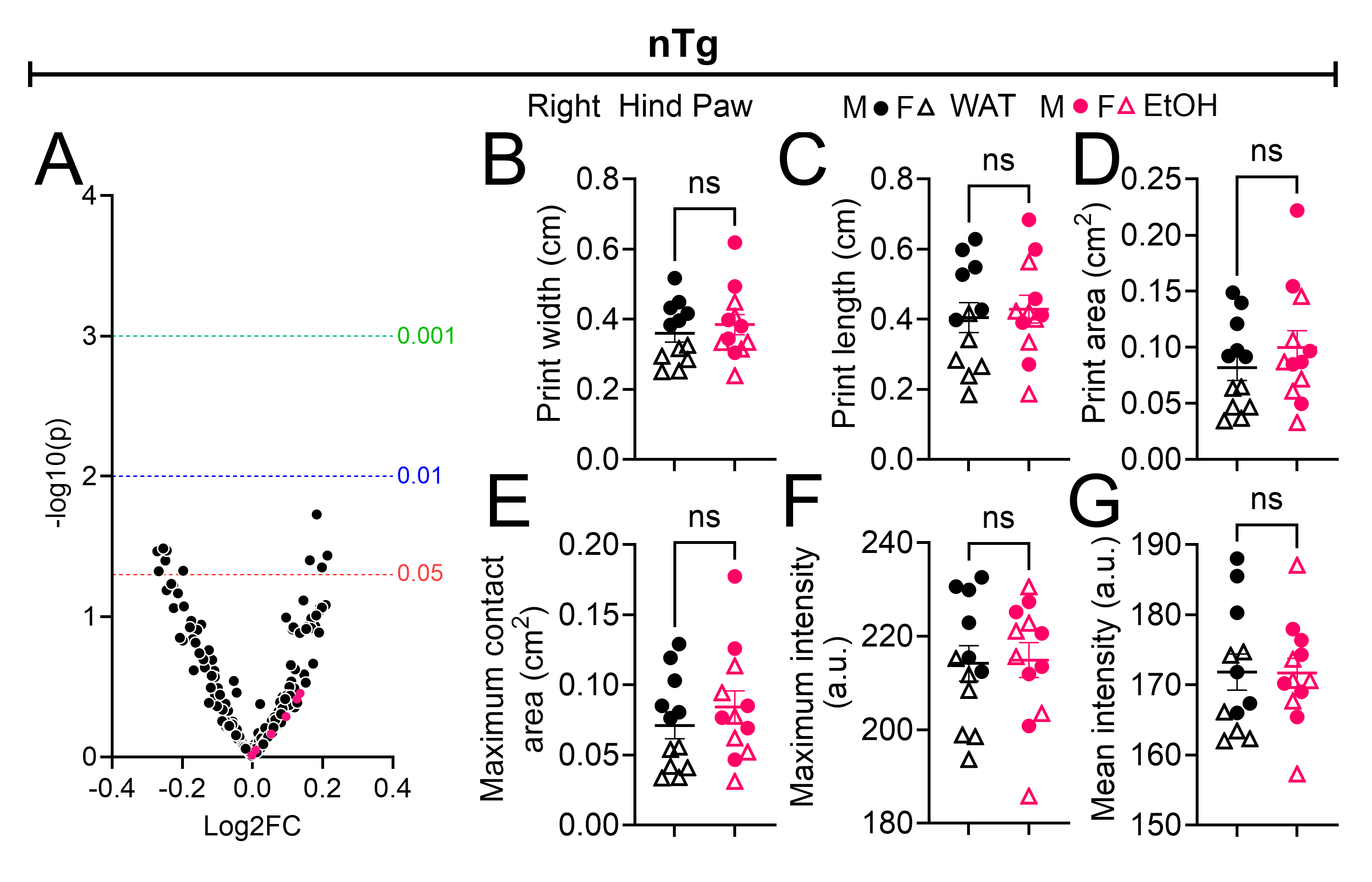

Supplement: Supporting Information: Figures [file NIHMS2142986-supplement-Supporting_Information__Figures.zip › acer70123-sup-0003-FigureS2@SUPPFIGURE_02.tif]

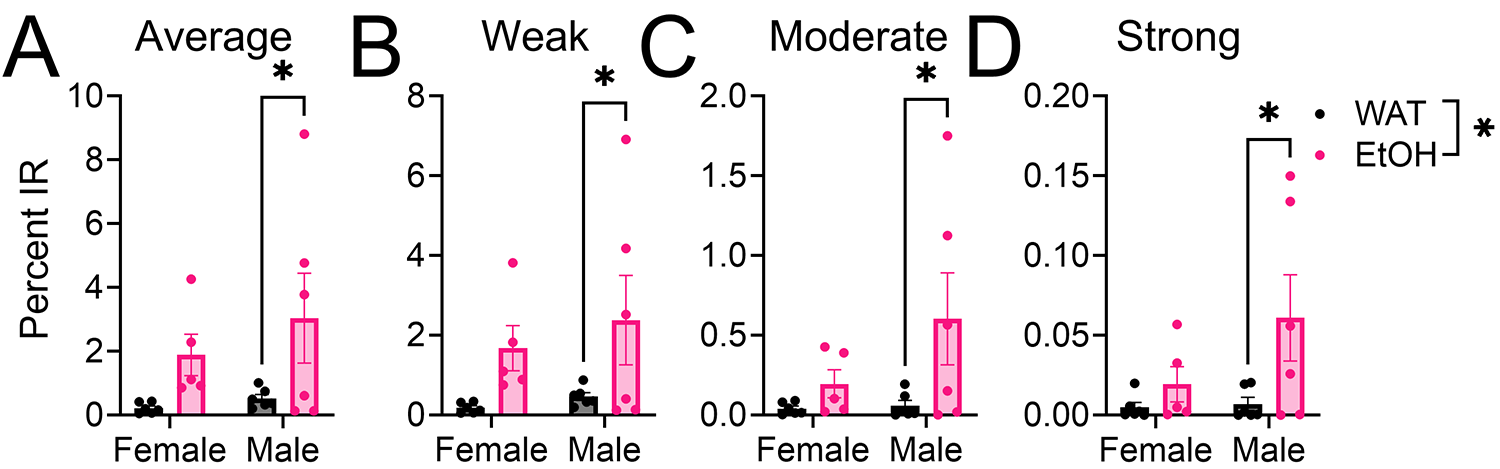

Supplement: Supporting Information: Figures [file NIHMS2142986-supplement-Supporting_Information__Figures.zip › acer70123-sup-0004-FigureS3@SUPPFIGURE_03_new.tif]

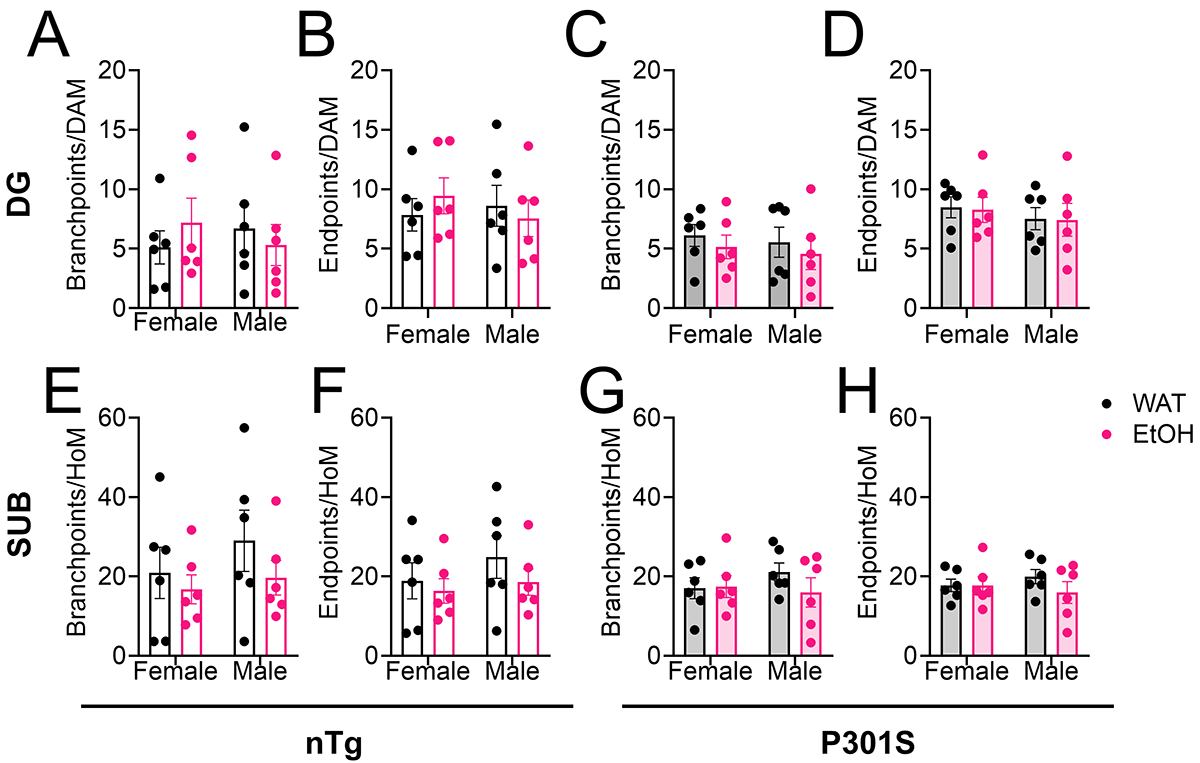

Supplement: Supporting Information: Figures [file NIHMS2142986-supplement-Supporting_Information__Figures.zip › acer70123-sup-0005-FigureS4@SUPPFIGURE_04_new.tif]
