# Supplementary material for: Alcohol consumption in P301S mice accelerates gait impairments, modifies aggregation of pathological tau and alters microglia within the hippocampus
Source: Alcohol Clin Exp Res (Hoboken). Author manuscript; Available in PMC 2026 Feb 25. (PMC12934799; doi:10.1111/acer.70123)
Supplement: Supporting Information: Table3 [file NIHMS2142986-supplement-Supporting_Information__Table3.docx]

**Supplementary Table 1. Primary and Secondary antibodies Used for IHC studies.**

| Antigen | Species | Dilution | Manufacturer | Cat. Number |
| --- | --- | --- | --- | --- |
| AT8 | Mouse | 1:500 | ThermoFisher | MN1020 |
| AT180 | Mouse | 1:500 | ThermoFisher | MN1040 |
| Iba1 | Rabbit | 1:500 | FUJIFILM/WAKO | 019-19741 |
| Secondary Antibody | | **Dilution** | **Manufacturer** | **Cat. Number** |
| Biotinylated AffiniPure^TM^ Goat Anti-Mouse IgG (H+L) | | 1:250 | Jackson ImmunoResearch Laboratories, Inc. | 115-065-003 |
| Biotinylated AffiniPure^TM^ Goat Anti-Rabbit IgG (H+L) | | 1:250 | Jackson ImmunoResearch Laboratories, Inc. | 111-065-003 |
