# Supplementary material for: Alcohol consumption in P301S mice accelerates gait impairments, modifies aggregation of pathological tau and alters microglia within the hippocampus
Source: Alcohol Clin Exp Res (Hoboken). Author manuscript; Available in PMC 2026 Feb 25. (PMC12934799; doi:10.1111/acer.70123)
Supplement: Supporting Information: Table2 [file NIHMS2142986-supplement-Supporting_Information__Table2.docx]

**Supplementary Table 2. pTau detection settings for typical slide, based on HALO® Digital Quantification of Image Analysis (v3.5.3) for AT8 and AT180.**

| Area Quantification Settings (AT8) | Avg. ± SD |
| --- | --- |
| weak intensity | 0.87 ± 0.30 |
| moderate intensity | 1.13 ± 0.27 |
| strong intensity | 1.44 ± 0.22 |
| Area Quantification Settings (AT180) | **Avg. ± SD** |
| weak intensity | 0.56 ± 0.13 |
| moderate intensity | 0.90 ± 0.11 |
| strong intensity | 1.29 ± 0.24 |
