# Supplementary material for: Alcohol consumption in P301S mice accelerates gait impairments, modifies aggregation of pathological tau and alters microglia within the hippocampus
Source: Alcohol Clin Exp Res (Hoboken). Author manuscript; Available in PMC 2026 Feb 25. (PMC12934799; doi:10.1111/acer.70123)
Supplement: Supporting Information: Table1 [file NIHMS2142986-supplement-Supporting_Information__Table1.docx]

**Supplementary Table 3.** **Microglia and Nucleus Detection Settings for typical slide, based on HALO® (v 3.6.4134.166) Microglia Activation FL Module (v1.0.6).**

| Microglia Detection | Settings |
| --- | --- |
| Cell Body Diameter | 3,20 |
| Min. Cell Body Intensity | 0.5 |
| Min. Process Intensity | 0.25 |
| Max Process Radius* | 30 |
| Max Fragmentation Length* | 8 |
| Activation Process Thickness* | 1.5 |
| Nucleus Detection | **Settings** |
| Nuclear Contrast Threshold | 0.517 |
| Min. Nuclear Intensity | 0.093 |
| Nuclear Size | 15.7643, 144.8071 |
| Min. Nuclear Roundness | 0.437 |
| Nuclear Segmentation Aggressiveness | 0.2 |
| Fill Nuclear Holes | False |
